# Stroke in COVID-19: A systematic review and meta-analysis

**DOI:** 10.1177/1747493020972922

**Published:** 2020-11-11

**Authors:** Stefania Nannoni, Rosa de Groot, Steven Bell, Hugh S Markus

**Affiliations:** 1 Stroke Research Group, Department of Clinical Neurosciences, University of Cambridge, UK; 2 Donor Medicine Research, Sanquin Research, Amsterdam, the Netherlands

**Keywords:** Stroke, COVID-19, SARS-CoV-2, acute cerebrovascular disease, hemorrhagic stroke

## Abstract

**Background:**

Coronavirus disease 2019 (COVID-19) has become a global pandemic, affecting millions of people. However, the relationship between COVID-19 and acute cerebrovascular diseases is unclear.

**Aims:**

We aimed to characterize the incidence, risk factors, clinical–radiological manifestations, and outcome of COVID-19-associated stroke.

**Methods:**

Three medical databases were systematically reviewed for published articles on acute cerebrovascular diseases in COVID-19 (December 2019–September 2020). The review protocol was previously registered (PROSPERO ID = CRD42020185476). Data were extracted from articles reporting ≥5 stroke cases in COVID-19. We complied with the PRISMA guidelines and used the Newcastle–Ottawa Scale to assess data quality. Data were pooled using a random-effect model.

**Summary of review:**

Of 2277 initially identified articles, 61 (2.7%) were entered in the meta-analysis. Out of 108,571 patients with COVID-19, acute CVD occurred in 1.4% (95%CI: 1.0–1.9). The most common manifestation was acute ischemic stroke (87.4%); intracerebral hemorrhage was less common (11.6%). Patients with COVID-19 developing acute cerebrovascular diseases, compared to those who did not, were older (pooled median difference = 4.8 years; 95%CI: 1.7–22.4), more likely to have hypertension (OR = 7.35; 95%CI: 1.94–27.87), diabetes mellitus (OR = 5.56; 95%CI: 3.34–9.24), coronary artery disease (OR = 3.12; 95%CI: 1.61–6.02), and severe infection (OR = 5.10; 95%CI: 2.72–9.54). Compared to individuals who experienced a stroke without the infection, patients with COVID-19 and stroke were younger (pooled median difference = −6.0 years; 95%CI: −12.3 to −1.4), had higher NIHSS (pooled median difference = 5; 95%CI: 3–9), higher frequency of large vessel occlusion (OR = 2.73; 95%CI: 1.63–4.57), and higher in-hospital mortality rate (OR = 5.21; 95%CI: 3.43–7.90).

**Conclusions:**

Acute cerebrovascular diseases are not uncommon in patients with COVID-19, especially in those whom are severely infected and have pre-existing vascular risk factors. The pattern of large vessel occlusion and multi-territory infarcts suggests that cerebral thrombosis and/or thromboembolism could be possible causative pathways for the disease.

## Introduction

In December 2019, several cases of unexplained pneumonia were diagnosed in Wuhan, China and then also diagnosed in other regions of the world, creating a global pandemic. Coronavirus disease 2019 (COVID-19) is caused by a severe acute respiratory syndrome (SARS)-like coronavirus (SARS-CoV-2). At the time of writing, the pandemic had affected more than 210 countries, with over 29 million confirmed cases and over 900,000 fatalities.^1^ In most patients, the disease is characterized by fever, dry cough, dyspnea, and hypoxia, with interstitial pneumonia features on chest X-ray or computed tomography scan.^2,3^ However, COVID-19 is not just a respiratory disease and can affect other organs, including the brain.

While several studies have highlighted a reduction in stroke admissions registered during the acute phase of the pandemic,^4^ there are accumulating reports of acute cerebrovascular disease (CVD) complicating COVID-19, including both acute ischemic stroke (AIS) and intracerebral hemorrhage (ICH).^5,6^ Previous reviews have shown an association between a past history of CVD and increased severity and mortality of COVID-19;^7,8^ others papers have reviewed the spectrum of neurological manifestation in COVID-19.^9–11^ However, whether COVID-19 may be considered a risk factor for stroke is still not established. Similarly, little is known about any specific characteristics of COVID-19-associated stroke.

We performed a systematic review and meta-analysis to investigate the relationship between COVID-19 and stroke. We used the data to answer the following questions: (1) What is the incidence of stroke in COVID-19 patients? (2) What are the risk factors for stroke in COVID-19 patients? (3) What are the characteristics of stroke in COVID-19 patients? (4) What is the outcome of stroke in COVID-19 patients? Finally, we discussed a range of the possible pathogenic mechanisms linking COVID-19 with stroke.

## Methods

### Search strategy and selection criteria

In this systematic review and meta-analysis, we searched published literature that provided evidence of acute cerebrovascular manifestations in COVID-19. The review protocol was registered before starting on PROSPERO (https://www.crd.york.ac.uk/PROSPERO/display_record.php?RecordID = 185476), and recommendations of the PRISMA statement were applied.^12,13^

Two medical (MEDLINE accessed from PubMed and Scopus) and one pre-prints (MedRxiv) databases were systematically reviewed for related articles from 1 December 2019 to 14 September 2020. In all electronic databases, our search criteria were based on predefined search terms (available in eMethods, Supplementary material). To ensure literature saturation, reference lists of included studies and relevant reviews identified through the search were scanned by the authors.

We included studies with information on new-onset cerebrovascular event(s) in patients with confirmed SARS-CoV-2 infection. Case reports and series, correspondence with relevant clinical data, case–control, and cohort studies were included for further review. We excluded studies that were reported as abstract-only (with no full-texts available), non-English articles, studies conducted on animal subjects, studies on pediatric populations, and repeat publications on the same patient cohorts.

Two authors (SN, RdG) participated in each phase of the review independently (screening, eligibility, and inclusion). They screened titles and abstracts, obtained full reports for all titles that appeared to meet the eligibility criteria or where there was any uncertainty, and decided whether these meet the inclusion criteria. All excluded studies were documented with reasons for exclusion. Any disagreement was resolved through consensus.

Case reports and observational studies were included in the quantitative analysis (meta-analysis) if they reported at least five cases of COVID-19 patients developing acute CVD. The list of the extracted variables for each included study was prespecified and is available in eMethods, Supplementary material. The quality assessment for each observational study included in the meta-analysis was performed using the adapted Newcastle–Ottawa Scale.^14^

### Data analysis

All analyses were performed using R v4.0.2. Median values of continuous traits were aggregated using the median of medians approach, and the median of the difference of the median between two groups was calculated via the metamedian package.^15,16^ To maximize the number of studies available for us to analyze, we also incorporated mean differences between two groups as simulations have shown that the median of the difference of medians method is robust when most of the studies considered report the sample median of the outcome.^16^ An approximate 95% confidence interval (95%CI) of the pooled value was calculated by inversing the sign test. Binary traits were analyzed using the meta package. We used a generalized linear mixed model to calculate the weighted average proportion, and 95%CIs for individual studies were calculated using the method proposed by Clopper and Pearson.^17^ Odds ratios (and their associated 95%CIs) were calculated and combined via random effect meta-analysis (inverse variance method) using the metabin function. Heterogeneity of effect sizes was quantified using I2 and Tau2 (DerSimonian-Laird estimator).

## Results

The literature search identified 2277 publications, including 770 from Pubmed, 1359 from Scopus, and 148 from MedRxiv; 371 were duplicates and removed. Therefore, 1906 unique papers were identified, and after abstract review, 193 were selected for full-text review (eFigure 1). Of these, 138 met inclusion criteria. Seven additional papers were identified from reference lists. Therefore, 145 papers were included in the systematic review; these comprised 57 case reports, 51 case series, 4 case–controls studies, and 33 cohort studies. Of these, 61 articles (24 case series, 4 case–controls, and 33 cohort studies) reported at least five stroke cases in COVID-19 patients and were included in meta-analysis. The complete reference list for the included papers is available in eTables 1 and 2. The quality assessment of the 33 included cohort studies is reported in eTable 3 and revealed a high quality in 14/33 (42%) records.

### Incidence of acute CVD in COVID-19 patients

**Figure 1.**
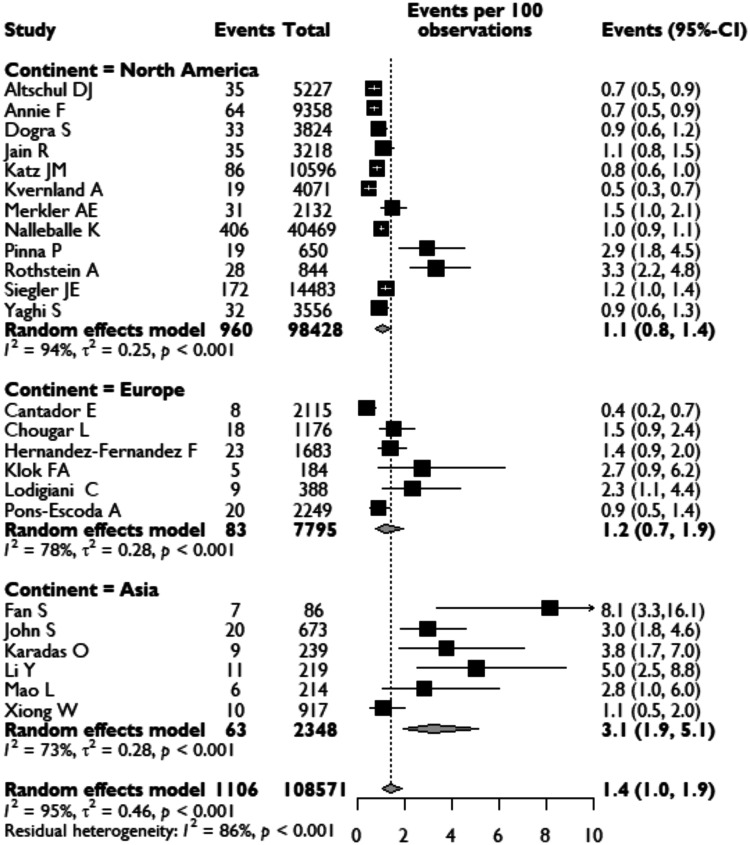
Pooled analysis of the proportion of COVID-19 patients developing acute CVD, presented for continents.

Twenty-four observational cohort studies reported the incidence of acute CVD in COVID-19 patients ranging from 0.4 to 8.1% (Figure 1). Across these studies, there were a total of 108,571 COVID-19 patients, of which ischemic or hemorrhagic stroke was reported in 1106 patients, yielding on meta-analysis an overall pooled incidence of acute CVD of 1.4% (95%CI: 1.0–1.9). These studies were conducted in different countries with varying ethnic demographics. Analysis of acute CVD incidence showed geographical variation, with a higher incidence reported in Asia (3.1%; 95%CI: 1.9–5.1) than in Europe (1.2%, 0.7–1.9) and North America (1.1%; 95%CI: 0.8–1.4) (Figure 1). We performed a sensitivity analysis, limiting the analysis to high-quality data only, which gave a similar pooled incidence of new CVDs in COVID-19 patients of 1.3% (95%CI: 0.9–1.8).

### Risk factors for stroke incidence in COVID-19 patients

**Figure 2.**
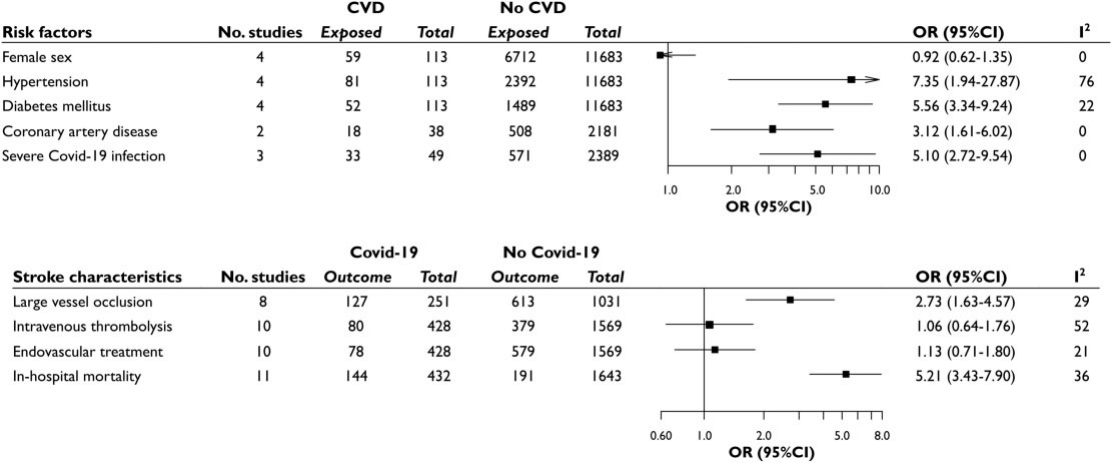
Risk factors for acute CVD in COVID-19 patients, showing the distributions of female sex, hypertension, diabetes, coronary artery disease, and severe COVID-19 in infected patients with and without stroke. Stroke characteristics of patients with and without COVID-19 are also showed, presenting the distribution of AIS from large vessel occlusion, the rates of acute stroke treatments, and of in-hospital deaths between the two groups.

Four studies were available to compare clinical characteristics of COVID-19 patients with CVD (*n* = 113) and without CVD (*n* = 11,683) (Figure 2 and Suppl. Figure e2). Compared with COVID-19 patients without CVD, COVID-19 patients that developed acute CVD were older (pooled median difference for age = 4.8 years; 95%CI = 1.7–22.4); there was no sex difference. Stroke risk in COVID-19 was higher in patients with cardiovascular risk factors, with patients developing CVD having greater likelihood of hypertension [81/113 vs 2392/11,683; OR = 7.35 (95%CI: 1.94–27.87)], diabetes mellitus [52/113 vs 1489/11,683; OR = 5.56 (95%CI: 3.34–9.24)], and coronary artery disease [18/38 vs 508/2181; OR = 3.12 (95%CI: 1.61–6.02)]. There was no significant difference in rates of smokers versus non-smokers [23/71 vs 560/9374; OR = 3.69 (95%CI: 0.47–29.23)]. Stroke in COVID-19 patients was associated with more severe infectious disease [33/49 vs 571/2389; OR = 5.10 (95%CI: 2.72–9.54)] (Figure 2 and Suppl. Figure e2). Both groups showed high level of D-dimer, without significant differences in median values (pooled median difference = 1248 µg/L; 95%CI: − 5600; 6400).

### Characteristics of COVID-19 patients developing acute CVD

Fifty studies were available for meta-analysis of clinical characteristics of COVID-19 patients with acute CVD. Demographics, vascular risk factors, COVID-19 characteristics, and blood investigations are presented in Table 1. Median age was 65.3 (61.4–67.6) years, and the majority was male (62.4%, 1141/1912). Vascular risk factors were common: hypertension (62.2%, 1111/1731), diabetes mellitus (36.7%, 612/1696), and dyslipidemia (25.2%, 625/947). The majority of patients manifested COVID-19 symptoms at stroke onset (84.1%, 350/453); the median delay of stroke from COVID-19 symptoms onset was 8.8 (6.3–11.6) days. When analyzing the clinical reason for admission (COVID-19 symptoms vs stroke symptoms), we found that neurological symptoms related to stroke represented the reason for hospital admission in 37.7% (414/1063) of patients. Sixty-one percent (609/1032) of patients suffered from a severe form of COVID-19; radiological signs of pneumonia were detected in 86.7% (198/246) patients and signs of pulmonary embolism in 14.8% (9/61). Laboratory investigations showed elevated median D-dimer (3720 µg/L) and fibrinogen (459 mg/L) levels. Information on antiphospholipid antibodies was available in 87 stroke cases; among these, 17.2% tested positive for IgM/IgG anti-cardiolipin or anti-β2-glycoprotein I antibodies.

**Table 1.** Demographics, clinical characteristics, laboratory variables, and outcome of COVID-19 patients developing acute CVD

Variable	*N* of valid studies	*N* of events	Pooled values
Demographics			
* *Age, years (median [95%CI])	50	1767	65.3 [60.4; 67.6]
* *Sex, female (% [95%CI])	50	771/1912	37.6 [33.2; 42.2]
Vascular risk factors			
* *Hypertension	45	1111/1731	62.2 [55.9; 68.1]
* *Dyslipidemia	37	625/947	25.2 [17.3; 35.1]
* *Diabetes mellitus	44	612/1696	36.7 [32.1; 41.6]
* *Atrial fibrillation	37	225/1326	13.9 [9.7; 19.5]
* *Smoking	35	246/1491	9.6 [5.9; 15.3]
* *Personal history of stroke	29	113/1314	8.0 [4.8; 13.0]
* *Coronary artery disease	30	254/1382	15.9 [12.1; 20.6]
Type of acute CVD			
* *Acute ischemic stroke	28	1329/1559	87.4 [80.1; 92.3]
* *Transient ischemic attack	28	15/1559	0.1 [0.0; 2.1]
* *Intracerebral hemorrhage	28	180/1559	11.6 [10.1; 13.3]
* *Cerebral venous thrombosis	28	25/1559	0.5 [0.1; 2.2]
COVID-19-related clinical variables			
* *COVID-19 symptoms present at stroke onset	32	350/453	84.1 [73.7; 91.0]
* *COVID-19 to stroke onset delay, day (median)	24	996	8.8 [6.3; 11.6]
* *Stroke as reason for admission	36	414/1063	37.7 [21.2; 57.6]
* *Severe disease	27	609/1032	60.5 [50.1; 70.0]
* *Intubation at stroke onset	10	7/66	2.9 [0.2; 35.1]
* *Pneumonia	14	198/246	86.7 [71.7; 94.3]
* *Pulmonary embolism	6	9/61	14.8 [7.9; 26.0]
Laboratory variables			
* *D-dimer, µg/L (median [95%CI])	29	937	3720 [1458; 5535]
* *Fibrinogen, mg/L (median [95%CI])	14	702	459 [361; 486]
* *Therapeutic anticoagulation at stroke onset	24	94/471	9.6 [4.5; 19.4]
* *Antiphospholipid antibodies positive	7	17/87	17.2 [7.0; 36.6]
* *Lupus anticoagulant positive	4	9/30	26.8 [5.5; 69.6]
Discharge outcomes			
* *In-hospital death	44	521/1655	31.5 [27.3; 36.0]
* *Discharged home	30	379/1315	19.1 [13.2; 26.8]
* *Discharged to rehabilitation	25	228/744	25.7 [18.9; 33.8]
* *Not discharged at time of publication	20	170/901	11.1 [4.7; 23.8]

Only studies reporting at least five patients with new-onset of CVD and COVID-19 were included in the pooled analysis.

Pooled values are presented as median (and 95% confident interval, CI) for continuous variables and as proportion (and 95%CI) for categorical variables.

### Stroke subtype, neuroimaging features, and outcome of COVID-19 patients developing acute CVD

We identified 1329 (87.4%) COVID-19 patients developing AIS and 180 (11.6%) ICH (Table 1). The median NIHSS score in patients with AIS was 15 (13–18), and a large vessel occlusion pattern of stroke was described in 79.6% (597/1189). Simultaneous involvement of different vascular territories in AIS was frequent (42.5%, 115/274) (Table 2). Details regarding acute stroke treatment were available for about 1200 patients with AIS: 19.1% (236/1205) received intravenous thrombolysis, whereas 25.9% (238/1223) underwent endovascular thrombectomy.

**Table 2.** Clinical and radiological characteristics of COVID-19 patients developing acute ischemic stroke and intracerebral hemorrhage

Variable	*N* of valid studies	*N* of events	Pooled values
Acute ischemic stroke in COVID-19			
Clinical variables			
* *NIHSS on admission (median [95%CI])	29	1202	15 [13–18]
* *Vigilance impairment at stroke onset	13	172/693	26.4 [14.6; 43.0]
Radiological variables			
* *Vascular territory, anterior	22	278/394	81.7 [70.2; 89.4]
* *Multiple infarction	15	115/274	42.5 [31.3; 54.5]
* * Large vessel occlusion	35	597/1189	79.6 [64.5; 89.3]
Acute stroke treatment			
* *Intravenous thrombolysis	34	236/1205	19.1 [12.4; 28.2]
* *Endovascular treatment—thrombectomy	36	238/1223	25.9 [13.5; 44.1]
* *Successful recanalization after thrombectomy	13	85/98	87.1 [76.2; 93.5]
Stroke etiology			
* *Cardioembolism	21	167/829	21.9 [16.5; 28.4]
* *Large artery atherosclerosis	20	112/819	10.6 [6.5; 16.8]
* *Small vessel disease	20	43/819	3.3 [1.3; 7.8]
* *Cryptogenic stroke	21	242/829	44.7 [27.1; 63.9]
Intracerebral hemorrhage in COVID-19			
* *Intraparenchymal hematoma, lobar	12	45/102	44.1 [34.7; 54.0]
* *Bilateral location	8	7/36	20.4 [8.2; 42.4]
* *Intracranial herniation	7	10/61	18.5 [6.5; 42.4]

Pooled values are presented as median (and 95% confident interval, CI) for continuous variables and as proportion (and 95%CI) for categorical variables.

Based on data from 829 cases, the most common stroke mechanism in AIS was cryptogenic (44.7%, 242/829), followed by cardioembolism (21.9%, 167/829) and large vessel atherosclerosis (10.6%, 112/819). Small artery stroke was infrequently reported (3.3%, 43/819).

Out of 102 patients with ICH, 44.1% showed a strictly lobar hematoma, and in 18.5% (10/61), the volume of hematoma led to intracranial herniation (Table 2).

Up to 44 studies reported data on discharge outcomes of patients with stroke and COVID-19 (Table 1). Out of the 1655 patients with information on mortality, 31.5% (521) suffered in-hospital death, whereas 19.1% (379/1315) were discharged home and 25.7% (228/744) were discharged to rehabilitation facilities.

### Stroke features in COVID-19 patients compared with non-COVID-19 patients with stroke

Eleven studies were analyzed to compare stroke characteristics in patients with and without COVID-19 (Suppl. Table e4). Patients with COVID-19 and stroke were younger than patients with stroke without infection (pooled median difference for age = −6.0; 95%CI = −12.3; −1.4), and female sex was less frequently affected [150/395 vs 773/1670; OR = 0.71 (95%CI: 0.51–0.99)]. Patients were less likely to have hypertension [257/385 vs 835/1128; OR = 0.65 (95%CI: 0.45–0.96)] and previous stroke [11/146 vs 159/720; OR = 0.34 (95%CI: 0.18–0.63)]; there was no significant difference in other cardiovascular risk factors (diabetes mellitus, dyslipidemia, smoking, coronary artery disease, and atrial fibrillation). AIS due to large vessel occlusion was more common in COVID-19 cases [127/251 vs 613/1031; OR = 2.73 (95%CI: 1.63–4.57)] (Figure 2). Stroke severity was higher in patients with stroke and COVID-19 (pooled median difference for NIHSS score 5; 95%CI = 3-9), and cryptogenic stroke was more common (26/41 vs 56/177; OR = 3.40, 95%CI: 1.16–10.00) (Suppl. Figure e3). Despite receiving acute stroke treatments (intravenous thrombolysis and thrombectomy) in similar proportions, individuals with stroke and COVID-19 infection showed higher in-hospital mortality (144/432 vs 191/1643; OR = 5.21, 95%CI: 3.43–7.90) (Figure 2).

## Discussion

In this systematic review and meta-analysis investigating the characteristics and outcomes of patients infected with SARS-CoV-2 and suffering a stroke, we found a pooled incidence of 1.4% of acute CVD in COVID-19. Individuals with COVID-19 who experienced concomitant stroke were more likely to be older and have pre-existing cardiovascular comorbidities and severe infection. Most patients had been admitted with COVID-19 symptoms, with stroke occurring a few days later. Ischemic stroke was the commonest stroke subtype and was frequently characterized by multiple cerebral infarctions and cryptogenic etiology. In comparison to strokes without COVID-19, people with CVD and COVID-19 were younger, suffered from more severe stroke, and stroke was more often caused by large artery occlusion.

There was variation in stroke incidence rates among individuals with COVID-19 across the included studies. This may reflect differences in the population studied; highest rates were reported in cohorts of critically ill patients^18,19^ and in studies analyzing neurological complications of COVID-19.^20–22^ It may also reflect differences in healthcare system organization and intensity of neurologic screening. Overall, we recorded the highest stroke rates in Asian populations.^18,23^ The severity of the infective disease consistently emerged as an important risk factor for stroke across different studies.^18,23,24^ Moreover, we found that people with COVID-19 developing a stroke were older than infected patients without stroke. This may partly explain the higher proportion of vascular risk factors that characterized the cerebrovascular group.

Comparison with non-infected patients with stroke showed that individuals with COVID-19 who developed stroke were significantly younger. There have been several reports on young patients without vascular risk factors admitted for large-artery stroke during the pandemic.^25–27^ Similarly, other studies highlighted a younger age of patients undergoing thrombectomy compared to the pre-pandemic period.^28–30^ Our pooled results confirmed these reports and suggest a particular profile of COVID-19-associated strokes, characterized clinically by severe NIHSS and poor outcome and radiologically by large artery occlusion and multiple arterial territory involvement. These strokes were more commonly labeled as cryptogenic compared to contemporary and historical stroke controls.^27^ These findings from the comparison between strokes with and without COVID-19 could suggest that some mechanisms directly related to COVID-19 have a role in the occurrence of stroke and explain the characteristic profile of stroke in infected patients. Large artery occlusion in COVID-19 may be primarily due to cardioembolism or paradoxical embolism and less often due to large artery atherosclerosis and plaque rupture,^31^ thus explaining the occurrence of stroke among young people without vascular risk factors, in individuals with high levels of D-dimer or other signs of hypercoagulability or in patients with pulmonary embolism and venous thrombosis.^19^

An important question is whether stroke occurring in individuals with COVID-19 is causally related or represents an incidental association due to COVID-19 infection being widespread in the community. The occurrence of stroke in those with COVID-19 does not provide direct evidence of causality between the two diseases. As with all observational studies, residual confounding may be an explanation, as a substantial proportion of the patients hospitalized with COVID-19 and stroke exhibit several vascular risk factors; also, some COVID-19-related factors, such as less-controlled vascular risk factors and mental stress, may contribute to stroke. However, a number of lines of evidence suggest that COVID-19 may be a trigger or risk factor for stroke at least in a proportion of cases. First, SARS-CoV-2 infection appears more likely to cause thrombotic vascular events, including stroke, than other coronavirus and seasonal infectious diseases, with a 7.6-fold increase in the odds of stroke with COVID-19 compared with influenza.^24^ Second, the characteristic pattern of stroke in individuals with COVID-19, with an increased proportion of large artery occlusion, infarction involving multiple territories, and increased cryptogenic etiology, suggests a causal relationship in at least a proportion of patients.

Previous reports have been published with the attempt to clarify the relationship between stroke and COVID-19. These included narrative reviews,^32,33^ systematic reviews,^9–11,34^ and meta-analyses.^34,35^ While some authors have focused on the relationship between personal history of CVD and COVID-19,^7,8^ we studied the incidence and risk factors for the occurrence of new CVD in concomitant SARS-CoV2 infection, searching for a specific profile of COVID-19-associated stroke. We found a similar rate of stroke incidence in COVID-19 compared to previous reports, but included a higher number of cohort studies.^34,35^ We provide a comprehensive picture of the clinical, biochemical, and radiological features of stroke in COVID-19; in comparison to other reviews,^36,37^ this was done after excluding case reports and small case series (which may be biased through focusing on particularly unusual cases), thus strengthening the solidity of our results. Core novelties of our investigation are synthesis of evidence on the topic of risk factors for stroke in people with COVID-19 as well as the comparison of stroke characteristics between infected and non-infected patients, both of which are vital to on-going clinical care and management during the current pandemic.

Our results may have important clinical implications. We demonstrated that stroke might complicate the course of COVID-19, with older and severely infected patients being at higher risk. Even if the incidence of stroke in COVID-19 population was less than 2%, the scale of the COVID-19 pandemic means that many thousands of people could potentially be affected by this complication globally. Therefore, clinicians should be vigilant for signs and symptoms of acute CVD in individuals with COVID-19 to ensure appropriate clinical interventions. Special attention should be paid in intubated or sedated patients, in whom awareness of potential neurological signs is important, for example by monitoring of Glasgow Coma Scale and pupil reaction, and in patients with abnormal elevation in coagulation laboratories or other thrombotic complications. Moreover, even if the majority of strokes occurred after a few days of COVID-19 symptoms onset, we found that neurological symptoms represented the reason of hospital admission in more than one-third of people with COVID-19 and stroke. These patients might have mild respiratory symptoms, or be completely asymptomatic, with subsequent important implications for stroke care re-organization. In fact, all patients with stroke in the pre-hospital setting should be treated as potential COVID-19 cases until the results of COVID-19 screening in the hospital are negative, and for patients with suspected or confirmed infection, a protected stroke pathway should be adopted.

### Mechanisms of stroke in individuals infected with COVID-19

**Figure 3.**
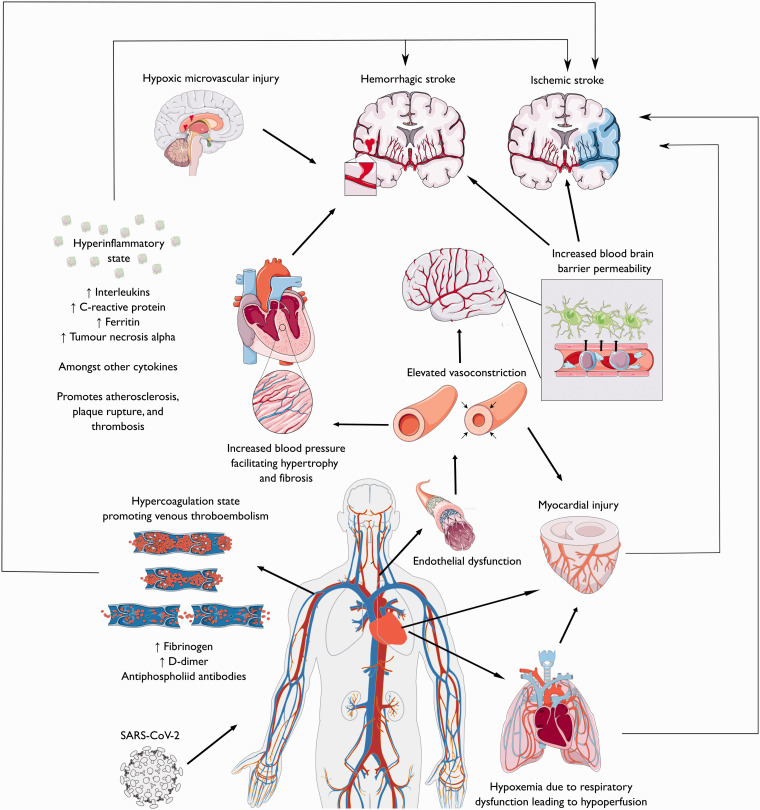
Overview on the possible stroke mechanisms in COVID-19 patients. This figure was created using Servier Medical Art templates, which are licensed under a Creative Commons Attribution 3.0 Unported License; https://smart.servier.com.

The mechanisms of cerebrovascular manifestations in people with COVID-19 are likely multifactorial. They could be related to conventional stroke mechanisms, with COVID-19 acting as a trigger.^38,39^ Alternatively, they could be directly caused by SARS-CoV-2 infection through specific pathophysiological mechanisms leading to both ischemic and hemorrhagic stroke (Figure 3).

### Ischemic stroke mechanisms in COVID-19

Activation of the coagulation pathway with elevated D-dimer and fibrinogen is a common feature of many individuals with severe COVID-19 infection. This *coagulopathy*, termed “sepsis-induced coagulopathy” (SIC), is related to the infection-induced systemic inflammatory response and may contribute to the increased risk of thrombosis and stroke.^40,41^ Also, the presence of antiphospholipid (aPL) antibodies, including IgA anticardiolipin antibodies and IgA and IgG beta 2 glycoprotein I antibodies, has been reported in severely infected patients with multiple cerebral infarcts.^23,42^ Hypercoagulation could lead to ischemic stroke promoting venous thromboembolism and paradoxical embolism; this could explain stroke from large vessel occlusion in young people without vascular risk factors, where plaque rupture or in situ thrombosis seems less likely.^31^

COVID-19, similar to other coronaviruses, uses the angiotensin-converting enzyme 2 (ACE-2) receptor to enter the cells.^43^ This receptor is expressed in the lungs, heart, kidneys, and vascular endothelium. Direct viral invasion of endothelial cells causes an inflammation or “endothelitis” which has been proposed as one of the substrates for the thrombotic complications of COVID-19.^44^ Moreover, binding of SARS-CoV-2 to ACE-2 receptor causes a depletion of its availability through endocytosis and ultimately a downregulation of the renin angiotensin system (RAS).^45^ In fact, the unopposed generation of Angiotensin II, no more counterbalanced by Angiotensin 1-7, worsens lung injury and is also responsible for *endothelial dysfunction* in organs like the heart and brain. This could result into increased sympathetic activity, loss of blood pressure autoregulation, and vasoconstriction with subsequent organ ischemia.^46^

The continuous and uncontrolled activation of the immune system caused by the viral infection, with subsequent excessive cytokine release or “*cytokine storm*,” has been implicated in brain damage during COVID-19. Cytokines/chemokines promote atherosclerosis, plaque rupture, and superimposed thrombosis.^47^ Together with endothelial injury, they can upregulate tissue factor expression and further promote a pro-thrombotic state.^48^

Various manifestations of *myocardial injury* have been described, including viral myocarditis*,* myocardial dysfunction related to the cytokine storm, coronary artery disease caused by oxygen supply and demand mismatch, and stress cardiomyopathy due to the stimulation of the sympathetic nervous system.^49,50^ All these mechanisms may lead to cardiac arrhythmias and intracardiac thrombus formation, possibly exacerbated by the hypercoagulable state, and could increase the risk of cardioembolic stroke.

Finally, some individuals with COVID-19 may be particularly susceptible to cerebrovascular injury from *hypoxemia.*^51^ In those with pre-existing intracranial stenosis, for example, hypoxemia could lead to infarction due to a mismatch between oxygen supply and demand.^52^ Similarly, cerebral *hypoperfusion* secondary to the downregulation of the RAS could increase the risk of both large vessel and SVD infarction, with a typical border-zone distribution.^53,54^

### Hemorrhagic stroke mechanisms in COVID-19

Our review highlighted that COVID-19-related hemorrhagic strokes are much less common than ischemic strokes. Whether the COVID-19 infection and intracerebral hemorrhage are casually related in these cases is unclear. However, some mechanisms mediating the increased risk of ischemic stroke in patients with COVID-19 could also play a role in promoting intracranial bleeding.^55,56^

The affinity of the SARS-CoV-2 for ACE2 receptors could allow the virus to directly damage intracranial arteries, causing vessel wall rupture. Also, *downregulation of RAS* may rise blood pressure and put patients already diagnosed with hypertension at higher risk for hemorrhagic stroke.^57^ Older individuals, affected by age-related ACE2 deficiency, might be particularly exposed to risk of ICH in this setting.

The *integrity of blood brain barrier* (BBB) could be impaired by the massive release of cytokines and proteases that accompanies the immune response to the SARS-CoV-2 infection.^57,58^ Besides ICH, the BBB breakdown could explain the cases of hemorrhagic posterior reversible encephalopathy syndrome (PRES) and hemorrhagic transformation of ischemic strokes that have been reported in some patients with COVID-19.^59^

Also, SARS-CoV-2 infection could be associated with a *consumption coagulop*athy related to fibrinogen depletion (either from metabolic acidosis or disseminated intravascular coagulation), which may increase the risk of ICH.^38^

Finally, perivascular micro-hemorrhages with cerebral microbleeds visible on susceptibility weighted MRI have been described in a few individuals with severe COVID-19 and neurologic complications.^60^ Their location in the corpus callosum and the subcortical and deep white matter was similar to the anatomical distribution seen in patients with hypoxic respiratory failure and sepsis, suggesting a potential role of *cerebral hypoxia* in causing brain injury in severe COVID-19.^60^

### Limitations

Given the recency of the pandemic, the findings from this review should be considered preliminary. Assumptions on the stroke incidence amongst people with COVID-19 were mostly based on small, single-center observational studies and therefore should be regarded with caution. Moreover, the number of cohort studies providing information on stroke control groups was limited, reducing the reliability of estimates of stroke risk in individuals infected with COVID-19. Also, the results on stroke etiologies and stroke outcome in COVID-19 might be affected by the fact that some patients were still hospitalized at the time of publication, which may limit the assessment of the natural course of the disease. Finally, we acknowledged that the meta-analysis results could be hampered by the heterogenous quality of the included papers, some of them rated as only moderate quality.

## Conclusions

We found 1.4% of individuals with COVID-19 suffered acute CVD. This risk was highest in those most severely infected and those with pre-existing vascular risk factors. The pattern of stroke differed from that in a non-COVID-19 stroke population. Most strokes were ischemic, and there was an increase in large vessel occlusion and multiple territory infarcts suggesting that increased thrombosis and thromboembolism could be important. Further studies are required to provide more robust estimates of the increase in stroke resulting from COVID-19 and to elucidate the precise pathophysiology linking COVID-19 to risk of CVD.

## Supplemental Material

sj-pdf-1-wso-10.1177_1747493020972922 - Supplemental material for Stroke in COVID-19: A systematic review and meta-analysis Click here for additional data file. Supplemental material, sj-pdf-1-wso-10.1177_1747493020972922 for Stroke in COVID-19: A systematic review and meta-analysis by Stefania Nannoni, Rosa de Groot, Steven Bell and Hugh S Markus in International Journal of Stroke
