# Supplementary material for: Stroke in COVID-19: A systematic review and meta-analysis
Source: Int J Stroke. 2020 Nov 11;16(2):137–49. doi: 10.1177/1747493020972922 (PMC7859578; doi:10.1177/1747493020972922)
Supplement: sj-pdf-1-wso-10.1177_1747493020972922 - Supplemental material for Stroke in COVID-19: A systematic review and meta-analysis [file sj-pdf-1-wso-10.1177_1747493020972922.pdf]

STROKE IN COVID-19: A SYSTEMATIC REVIEW AND META-ANALYSIS

Supplementary material

Stefania Nannoni, MD PhD[1], Rosa de Groot, MSc[2], Steven Bell, PhD[1], Hugh S Markus, DM, FMedSci[1]

1. Stroke Research Group, Department of Clinical Neurosciences, University of Cambridge, UK
2. Donor Medicine Research, Sanquin Research, Amsterdam, the Netherlands

eMethods. Search terms and variables definition
eTable 1. Study information and number of participants of each study on acute cerebrovascular diseases and COVID-19 included in the systematic review (but not in the meta-analysis).
eTable 2. Study information, number of participants and main findings of each study on acute cerebrovascular diseases and COVID-19 entered in the meta-analysis.
eTable 3. Quality assessment of the cohort studies included in the meta-analysis based on the Newcastle-Ottawa scale.
eTable 4. Clinical characteristics of cerebrovascular disease (CVD) patients with or without COVID-19.
eFigure 1. PRISMA flow diagram based on literature search updated at 05.08.2020.
eFigure 2. Clinical characteristics of COVID-19 patients with and without new onset of acute CVD.
eFigure 3. Distrubution of stroke etiologies in stroke patients with and without COVID-19.
eReferences (n=148)

eMethods.

Search terms: ((((COVID 19) OR COVID19 OR (COVID-19) OR (2019 nCoV) OR 2019nCoV OR (2019-nCoV) OR (2019 novel coronavirus) OR (new coronavirus) OR (novel coronavirus) OR (SARS CoV-2) OR (SARS-CoV-2) OR (SARS-CoV) OR (Wuhan AND coronavirus)))) AND (((Stroke OR (acute cerebrovascular disease) OR (brain infarction) OR (intracranial hemorrhage) OR (cerebral hemorrhage) OR ICH OR (cerebral thrombosis) OR (cerebral venous thrombosis)))

Variables definition: For each study included in the quantitative analysis, we extracted the following variables:

1. Study information: study design and country
2. Number of included patients: including stroke patients with COVID-19, and eventually COVID-19 control group (i.e. COVID-19 patients who did not have stroke) and stroke control group (i.e. contemporary or historical stroke patients without COVID-19).
3. Demographic variables: age, and proportion of female.
4. Vascular risk factors: History of hypertension, dyslipidemia, diabetes mellitus, atrial fibrillation, active smoking, coronary artery disease, of prior stroke or transient ischemic attack (TIA).
5. Stroke subtypes: acute ischemic stroke, TIA, intracerebral hemorrhage (ICH), cerebral venous thrombosis (CVT).
6. Stroke-related clinical variables: admission National Institutes of Health Stroke Scale (NIHSS) score for AIS patients (median value); vigilance impairment at stroke onset for AIS patients; delay since COVID-19 symptoms onset to stroke onset (days, median value); reason for hospital admission (stroke vs COVID-19).
7. COVID-19-related clinical variables: presence of COVID-19 symptoms at stroke onset; severity of COVID-19 (severe(1) vs not severe), intubation at stroke onset, radiological signs of pneumonia, radiological signs of pulmonary embolism.
8. Laboratory variables: plasmatic D-dimer level (µg/L, level closest to the time of the stroke median value), plasmatic fibrinogen level (mg/L, median value), lupus anticoagulant, anti-phospolipidies antibodies, therapeutic anticoagulation at the time of stroke onset.
9. Imaging variables for AIS patients: vascular territory involved (anterior vs posterior circulation), infarct pattern (multiple vs single vascular territories), presence of proximal large artery occlusion (defined as occlusion of the intracranial internal carotid artery, M1, and M2 segments of the middle cerebral artery, basilar artery, or P1 segment of the posterior cerebral artery).

10. Imaging variables for ICH patients: location of intraparenchymal hematoma (lobar vs not lobar), bilateral location, ICH leading to intracranial herniation.
11. Acute stroke in-hospital treatment: number of patients undergoing intravenous thrombolysis (IVT), endovascular treatment (EVT), and number of successful recanalization after EVT (defined as TICI score=2b-3)(2).
12. Stroke etiology: classified based on the Trial of ORG 10172 in Acute Stroke Treatment classification(3), including: large artery atherosclerosis, cardioembolism, small vessel disease, other determined mechanism (defined as an alternative mechanism such as dissection or known hypercoagulability, excluding de novo hypercoagulability in the setting of a COVID-19) and cryptogenic (defined as cases not meeting criteria for any of the above stroke subtypes, including those with incomplete workup or with multiple competing high-risk mechanisms).
13. Discharge outcomes: in-hospital mortality, discharge disposition (home, rehabilitation or not discharged at the time of publication).

eTable 1. Study information and number of participants of each study on acute cerebrovascular diseases and COVID-19 included in the systematic review (but not in the meta-analysis).

First author (ref)	Country	Study design	COVID-19 patients with acute CVD (n)	Patients with AIS (n)	Patients with ICH (n)
Agarwal A(4)	India	Cohort study	2	0	2
Al Saiegh F(5)	USA	Case series	2	1	0
Argirò R(6)	Italy	Case series	6	0	0
Avula A(7)	USA	Case series	4	4	0
Baldacini M(8)	France	Case report	1	1	0
Barrios-López JM(9)	Spain	Case series	2	2	0
Baudar C(10)	Belgium	Case report	1	0	0
Bigliardi G(11)	Italy	Case report	1	1	0
Bolaji P(12)	UK	Case report	1	0	0
Bonardel C(13)	France	Case report	1	1	0
Brüggemann B(14)	Netherlands	Case report	1	1	0
Burkert J(15)	UK	Case report	1	1	0
Carroll E(16)	USA	Case series	2	0	2
Castillo PR(17)	USA	Case series	2	0	2
Cavalcanti DD(18)	USA	Case series	3	0	0
Cavallieri F(19)	Italy	Case report	1	1	0
Chen W(20)	China	Case report	1	1	0
Chibane S(21)	Canada	Case report	1	1	0
Chougar L(22)	France	Case report	1	0	0
Co C(23)	Philippines	Case report	1	1	0
Deliwala S(24)	USA	Case report	1	1	0
Diaz-Segarra N(25)	USA	Case series	4	4	0
Duroi I(26)	Belgium	Case report	1	1	0
Fara MG(27)	USA	Case series	3	3	0
Frisullo G(28)	Italy	Case report	1	1	0
Garaci F(29)	Italy	Case report	1	0	0
Garg A(30)	USA	Case report	1	1	0
Gemcioglu E(31)	Turkey	Case report	1	1	0
Ghani MU(32)	USA	Case series	3	0	3
Gill I(33)	USA	Case report	1	1	0
Goldberg MF(34)	USA	Case report	1	1	0
González-Pinto T(35)	Spain	Case report	1	1	0
Griffin D(36)	USA	Case report	1	1	0
Guillan M(37)	Spain	Case report	1	1	0
Gulko E(38)	USA	Case series	2	2	0
Gunasekaran K(39)	USA	Case report	1	1	0
Helms J(40)	France	Cohort study	4	3	1
Helms J(41)	France	Cohort study	3	3	0

Heman-Ackah SM(42)	USA	Case series	2	0	2
Hemasian H(43)	Iran	Case report	1	0	0
Hoelscher C(44)	USA	Case report	1	0	0
Hossri S(45)	USA	Case report	1	1	0
Hughes C(46)	UK	Case report	1	0	0
Jensen MP(47)	UK	Case series	1	1	0
Kariyanna PT(48)	USA	Case report	1	1	0
Khan AW(49)	Pakistan	Case report	1	1	0
Klein DE(50)	USA	Case report	1	0	0
Li J(51)	China	Case report	1	0	1
Lima CFC(52)	Brasil	Case report	1	1	0
Llansó L(53)	Spain	Case report	1	0	0
Mahboob S(54)	USA	Case report	1	1	0
Maldonado S(55)	Belgium	Case report	1	1	0
Malentacchi M(56)	Italy	Case report	1	1	0
Moshayedi P(57)	USA	Case report	1	1	0
Motoie R(58)	Japan	Case report	1	0	1
Muhammad S(59)	Germany	Case report	1	0	1
Nicholson P(60)	Canada	Case series	4	0	4
Papi C(61)	Italy	Case report	1	1	0
Poillon G(62)	France	Case series	2	0	0
Radmard S(63)	USA	Case series	4	3	1
Rinkel LA(64)	Netherlands	Cohort study	7	7	0
Roy-Gash F(65)	France	Case report	1	0	0
Rudilosso S(66)	Spain	Case report	1	1	0
Saggese CE(67)	Italy	Case report	1	1	0
Salahuddin H(68)	USA	Case report	1	1	0
Sangalli D(69)	Italy	Case series	4	4	0
Scullen T(70)	USA	Cohort study	4	4	0
Seabra C(71)	Portugal	Case report	1	1	0
Sharifi-Razavi A(72)	Iran	Case report	1	0	1
Sharifi-Razavi A(73)	Iran	Case series	3	3	0
Sparr SA(74)	USA	Case series	4	4	0
Trifan G(75)	USA	Case report	1	0	1
Tunc A(76)	Turkey	Case series	4	4	0
Usman AA(77)	USA	Case series	3	0	3
Valderrama EV(78)	USA	Case report	1	1	0
Viguier A(79)	France	Case report	1	1	0
Vu D(80)	USA	Case report	1	1	0
Wee NK(81)	Singapore	Case report	1	0	1
Williams OH(82)	UK	Case report	1	1	0
Zahid MJ(83)	USA	Case report	1	0	1
Zayet S(84)	France	Case series	2	2	0
Zhai P(85)	China	Case report	1	1	0

Zhang Y(86)	China	Case series	3	3	0
Zhou B(87)	China	Case report	1	1	0

eTable 2. Study information, number of participants and main findings of each study on acute cerebrovascular diseases and COVID-19 entered in the meta-analysis. We also reported if each included study was suitable for the four main aims of our meta-analysis, with 1) indicating stroke incidence, 2) risk factors for stroke, 3) stroke features and 4) clinical outcome of COVID-19-associated stroke.

First author (ref)	Country	Study design	COVID-19 patients (n)	COVID-19 patients with acute CVD (n)	Patients with AIS (n)	Patients with ICH (n)	Age, median	Sex, F (n)	COVID-19 symptoms at stroke onset	COVID-19 to stroke onset, median (d)	NIHSS, median	LVO	Control group(s)	Aims for meta-analysis
Al Kasab S(88)	Usa	Retrospective observational		13	13		58	5			19	13	445 stroke cases without COVID-19	3; 4
Altschul DJ (b)(89)	USA	Retrospective observational		13	13			8			16	13	23 stroke cases without COVID-19	3; 4
Altschul DJ(90)	USA	Retrospective observational	5227	35		35	67	14						1; 3
Annie F(91)	USA	Retrospective observational	9358	64	64			40					9294 COVID-19 patients without stroke	1; 2; 3
Ashrafi F(92)	Iran	Case series		6	6	0	43.5	3	6		10	6		3
Belani P(93)	USA	Case control		19	19									3
Benger M(94)	UK	Case series		5		5	52.2	2	5	32				3
Benussi A(95)	Italy	Retrospective observational		43	35	3	76.9	21			10		68 stroke cases	3; 4

													without COVID-19	
Berekashvili K(96)	USA	Case series		10	10		55	6	10	5.5	13	5		3
Beyrouti R(97)	UK	Case series		6	6		68.5	1	5	11.5		6		3
Cantador E(98)	Spain	Case series	2115	8	7	0	76.4	1	8	6.3	4	4		1; 3
Cappellari M(99)	Italy	Case series		47	47		77	15		5	12	34		3
Chougar L(100)	France	Retrospective observational	1176	18	17	0								1
D'Amore F(101)	Italy	Case series		6	5	1	75.5	3				2		3
D'Anna L(102)	UK	Case series		8	7	1	74	1	8	7	8.5	3		3
Dmytriw A(103)	USA	Case series		69	69			30	61			31		3
Dogra S(104)	USA	Retrospective observational	3824	33		33	62	7		17				1; 3
Escalard S(105)	France	Retrospective observational		10	10		59.5	2	8	6	22	10	27 stroke cases without COVID-19	3; 4
Fan S(106)	China	Retrospective observational	86	6	6	0	68.2	1	6				80 COVID-19 patients without stroke	1; 2; 3

Hernández-Fernández F(107)	Spain	Retrospective observational	1683	23	17	5		6	12	5	16	10		1; 3; 4
Immovilli P(108)	Italy	Case series		19	17	2	76	9	15					3
Jain R(109)	USA	Retrospective observational	3218	35	26	9	66					17		1; 3
Jillella DV(110)	USA	Case series		8	8		64	3		2				3
John S(111)	United Arab Emirates	Retrospective observational	673	20	15		46.5	1	9		21.5	15		1; 3
Karadas O(112)	Turkey	Retrospective observational	239	9	4	2								1
Katz JM(113)	USA	Retrospective observational	10596	86	72	14		38				49	499 stroke cases without COVID-19	1; 3; 4
Khan M(114)	Arab Emirates	Case series		22	22		46.3	2	16	12.5		16		3
Kihira S(115)	USA	Case-control		126	126			49				40	203 stroke cases without COVID-19	3
Klok FA(116)	Netherla nds	Retrospective observational	184	5	5				5					1; 3
Kremer S(117)	France	Retrospective observational		17	17		75	6	5			8		3
Kvernland A(118)	UsA	Retrospective observational	4071	19		19	60	4					44 stroke cases without COVID-19	1; 3; 4

Lapergue B(119)	France	Case series		6	6		52	2	6	11.5	20	6		3
Li Y(120)	China	Retrospective observational	221	13	11	1	73.5	6	7	12			208 COVID-19 patients without stroke	1; 2; 3
Liang JW(121)	USA	Case series		7	7		57	1	7		18	7		3
Lin C(122)	Usa	Retrospective observational		9	9			6					51 stroke cases without COVID-19	3; 4
Lodigiani C(123)	Italy	Retrospective observational	388	9	9		71	3	3					1; 3
Majidi S(124)	USA	Case-control		24	24			5	14		13	24	21 stroke cases without COVID-19	3
Mao L(125)	China	Retrospective observational	214	6	5	1			4	9				1; 3
Mehrpour M(126)	Iran	Retrospective observational		10	9	1		5			20	7	120 stroke cases without COVID-19	3; 4
Meppiel E(127)	France	Case series		63	52	5	65	23		12		16		3
Merkler AE(128)	USA	Retrospective observational	2132	31	31		69	13			16		2101 COVID-19 cases without stroke	1; 2; 3

Mohamud AY(129)	USA	Case series		6	6		65.8	1	4	5.5	24.5	4		3
Morassi M(130)	Italy	Case series		6	4	2	69	1	4	19.5				3
Nalleballe K(131)	USA	Retrospective observational	40469	406	406	0								1
Nawabi J(132)	Germany	Case series		18		18	49.5	9	16	11				3
Ntaios G(133)	Greece	Retrospective observational		174	174		71.2	66		7	10		174 stroke cases without COVID-19	3; 4
Oxley TJ(134)		Case series		5	5		41	1	3		17	5		3
Paterson RW(135)	UK	Retrospective observational	43	8	8	0	62.5	2		8		4		3
Pinna P(136)	USA	Retrospective observational	650	19	10	8								1; 3
Pons-Escoda A(137)	Spain	Retrospective observational	2249	20	13	7	71	7	20			8		1; 3
Pop R(138)	France	Case series		13	13		78	8	4		13	13		3
Reddy ST(139)	USA	Case series		12	10	2	56.5	6	11	7	11			3
Rothstein A(140)	USA	Retrospective observational	844	28	20	8	60.5	12	28	23		3		1; 3

Shahjouei S(141)	USA	Retrospective observational		432	323	68	68	183		2.3	9	126		3
Siegler JE(142)	USA	Retrospective observational	14483	172	156	28		68			13	57		1; 3
Sweid A(143)	USA	Case series		22	17	3	59.5	12		8.8	14	15		3
Varatharaj A(144)	UK	Prospective observational	125	66	57	9	71	44						3
Wang A(145)	USA	Case series		5	5		52.8	1			27	5		3
Xiong W(146)	China	Retrospective observational	917	10	10	0			10					1; 3
Yaeger KA(147)	USA	Case series		5	5				3			5		3
Yaghi S(148)	USA	Retrospective, cohort study	3556	32	32		63	9	27	10	19	10	126 stroke cases without COVID-19	1; 3; 4

Table e3. Quality assessment of the cohort studies included in the meta-analysis based on the Newcastle-Ottawa scale.

Paper	Selection				Comparability	Outcome			SCORE
First author[ref]	**Representati-veness of the exposed cohort**	**Selection of the non exposed cohort**	**Ascertainment of exposure**	**Demonstration that outcome of interest was not present at start of study**	**Comparability of cohorts on the basis of the design or analysis**	**Assessment of outcome**	**Was Follow-Up Long Enough for Outcomes to Occur**	**Adequacy of Follow Up of Cohorts**	**(quality)**
Al Kasab S(88)	*	*	*	*	**	*	*	*	9 (high)
Altschul DJ (b)(89)	*	*	*	*	**	*	*	*	9 (high)
Altschul DJ(90)	*	*	*	*		*	*	*	7 (fair)
Annie F(91)	*	*	*	*	**	*	*	*	9 (high)
Benussi A(95)	*	*	*	*	**	*	*	*	9 (high)
Chougar L(22)	*	*	*	*		*	*		6 (moderate)
Dogra S(104)	*	*	*	*		*	*	*	7 (fair)
Escalard S(105)	*	*	*	*	**	*	*	*	9 (high)
Fan S(106)	*	*	*	*	**	*	*	*	9 (high)
Hernández-Fernández F(107)	*	*	*	*	**	*	*	*	9 (high)
Jain R(109)	*	*	*	*		*	*	*	7 (fair)
John S(111)	*		*	*		*	*	*	7 (fair)
Karadas O(112)	*	*	*	*		*	*		6 (moderate)

Katz JM(113)	*	*	*	*	**	*	*	*	9 (high)
Klok FA(116)	*	*	*	*		*	*		6 (moderate)
Kremer S(117)	*		*	*		*	*		5 (moderate)
Kvernland A(118)	*	*	*	*	**	*	*	*	9 (high)
Li Y(120)	*	*	*	*	**	*	*	*	9 (high)
Lin C(122)	*	*	*	*	**	*	*	*	9 (high)
Lodigiani C(123)	*	*	*	*		*	*	*	7 (fair)
Mao L(125)	*	*	*	*		*	*		6 (moderate)
Mehrpour M(126)	*	*	*	*	**	*	*	*	9 (high)
Merkler AE(128)	*	*	*	*	**	*	*	*	9 (high)
Nalleballe K(131)	*	*	*	*		*	*		6 (moderate)
Paterson RW(135)	*	*	*	*		*	*	*	7 (fair)
Pinna P(136)	*	*	*	*		*	*		6 (moderate)
Pons-Escoda A(137)	*	*	*	*		*	*		6 (moderate)
Rothstein A(140)	*	*	*	*		*	*	*	7 (fair)
Shahjouei S(141)	*		*	*		*	*	*	6 (moderate)
Siegler JE(142)	*		*	*		*	*	*	7 (fair)
Varatharaj A(144)	*		*	*		*	*		5 (moderate)
Xiong W(146)	*	*	*	*		*	*	*	7 (fair)

Yaghi S(148)	*	*	*	*	**	*	*	*	9 (high)

Newcastle-Ottawa scale for cohort studies:

Note: A study can be awarded a maximum of one star for each numbered item within the Selection and Outcome categories. A maximum of two stars can be given for Comparability. Blank: information not available, therefore not granted a star

Selection

1) Representativeness of the exposed cohort

a) truly representative of the average __of COVID-19 patients__ (describe) in the community ✸ (eg, consecutively admitted COVID-19 patients at one hospital)

b) somewhat representative of the average _______________ in the community ✸

c) selected group of users eg nurses, volunteers

d) no description of the derivation of the cohort

2) Selection of the non exposed cohort

a) drawn from the same community as the exposed cohort ✸ (information of COVID-19 patients without stroke or non-infected stroke patients during the same study period)

b) drawn from a different source

c) no description of the derivation of the non exposed cohort

3) Ascertainment of exposure

a) secure record (eg surgical records) ✸ (confirmation of SARS-CoV-2 infection)

b) structured interview ✸

c) written self report

d) no description

4) Demonstration that outcome of interest was not present at start of study

a) yes ✸ (new acute CVD on hospital admission or during the hospitalization in COVID-19 patients, excluding personal history of previous stroke)

b) no

Comparability

1) Comparability of cohorts on the basis of the design or analysis

a) study controls for ______age_________ (select the most important factor) ✸ (comparison between stroke and non-stroke patients with COVID-19; or comparison between stroke patients with and without COVID-19)

b) study controls for any additional factor ✸ (This criteria could be modified to indicate specific control for a second important factor. Any VRF)

Outcome

1) Assessment of outcome (i.e.occurrence of acute CVD in COVID-19 patients)

a) independent blind assessment ✸

b) record linkage ✸

c) self report

d) no description

2) Was follow-up long enough for outcomes to occur

a) yes (select an adequate follow up period for outcome of interest) ✸

b) no

3) Adequacy of follow up of cohorts

a) complete follow up - all subjects accounted for at least discharge modalities ✸

b) subjects lost to follow up unlikely to introduce bias - small number lost - > _10% (select an adequate %) follow up, or description provided of those lost) ✸

c) follow up rate < ____% (select an adequate %) and no description of those lost

d) no statement

Table e4. Clinical characteristics of cerebrovascular disease (CVD) patients with or without COVID-19.

Variable	N of valid studies	CVD patients with COVID-19	CVD patients without COVID-19	OR (95% CI) or pooled median
Demographics				
Age, median	11	590	1194	-6.0 (-12.3;-1.4)*
Sex, female	11	150/395	773/1670	0.71 (0.51;0.99)*
Vascular risk factors				
Hypertension	10	257/385	835/1128	0.65 (0.45;0.96)*
Hypercholesterolemia	10	140/385	471/1128	0.84 (0.65;1.08)
Diabetes mellitus	10	152/385	248/1128	1.66 (0.97;2.85)
Atrial fibrillation	9	73/342	108/1060	1.13 (0.32;4.06)
Smoking	8	56/342	146/1063	0.77 (0.41;1.45)
Coronary artery disease	5	39/234	72/496	1.05 (0.50;2.20)
Previous stroke	4	11/146	159/720	0.34 (0.18;0.63)*
Stroke characteristics				
AIS from LVO	8	127/251	613/1031	2.73 (1.63;4.57)*
NIHSS for AIS patients	10	477	1204	5 (3;9)*
AIS in anterior circulation	4	55/60	463/516	1.12 (0.36;3.51)
Acute stroke treatment				
IVT	10	80/428	379/1569	1.06 (0.64;1.76)
EVT	10	78/428	579/1569	1.13 (0.71;1.80)
Successful recanalization after EVT	3	24/26	399/481	1.48 (0.30;7.29)
Discharge outcomes				
In-hospital death	11	144/432	191/1643	5.21 (3.43;7.90)*

*denotes significant result

Legend: AIS= acute ischemic stroke; LVO= large vessel occlusion; IVT= intravenous thrombolysis; EVT=endovascular treatment.

Figure e1. PRISMA flow diagram based on literature search at 14.09.2020.

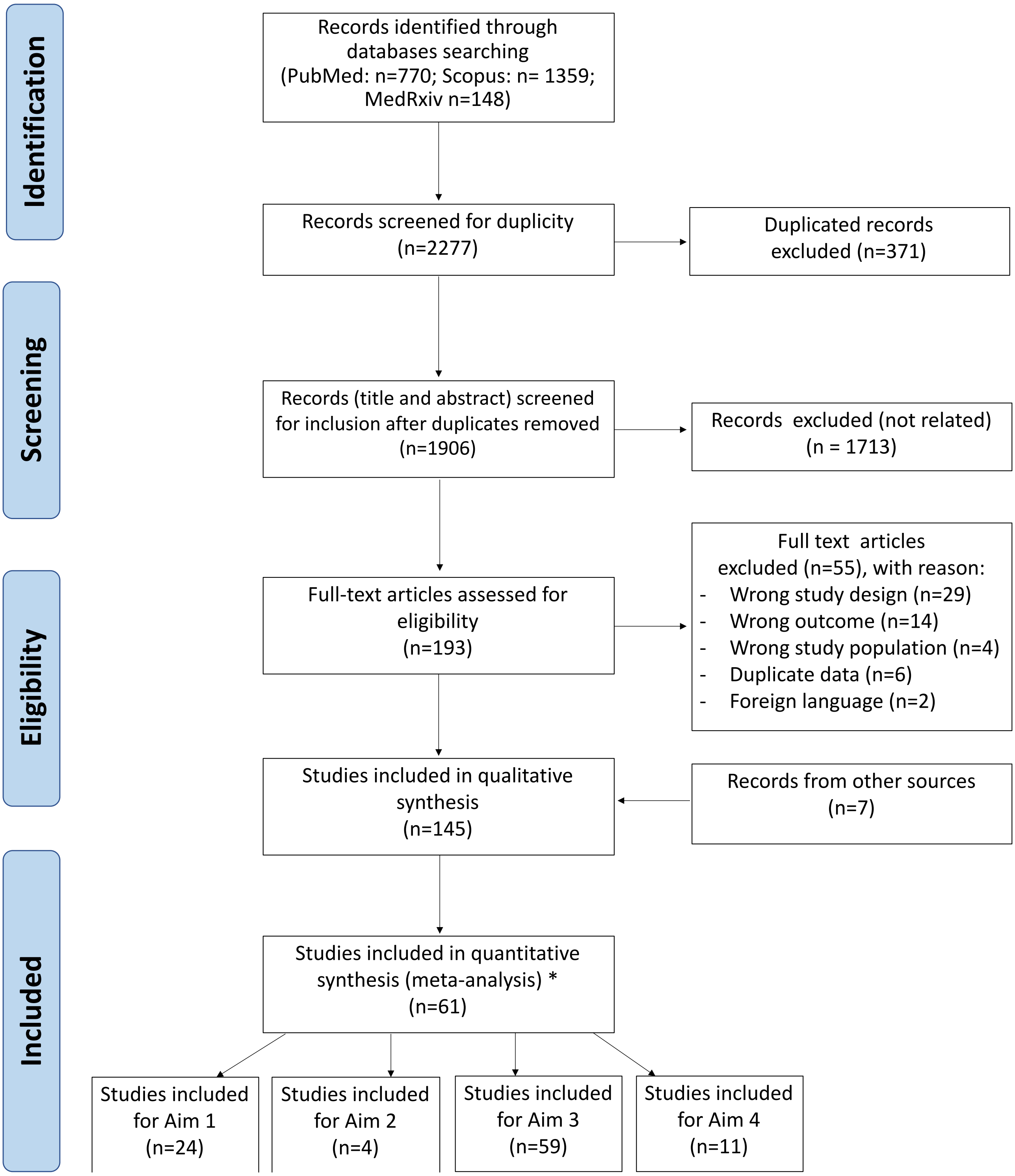

Legend: * After excluding articles reporting less than 5 cases stroke in COVID-19.
Aim 1) indicates stroke incidence, 2) risk factors for stroke, 3) stroke features and 4) clinical outcome of COVID-19-associated stroke.

Figure e2. Clinical characteristics of COVID-19 patients with (CVD group) and without new onset of acute CVD (No CVD group). Figure 2a reports distribution of female sex (Sex_F); Figure 2b proportions of hypertension (HTN); Figure 2c proportions of diabetes mellitus (DM); Figure 2d proportions of coronary artery disease (CAD); Figure 2e proportions of smoking; and Figure 2f proportions of severe COVID-19 (COVID severe).

Figure 2a.

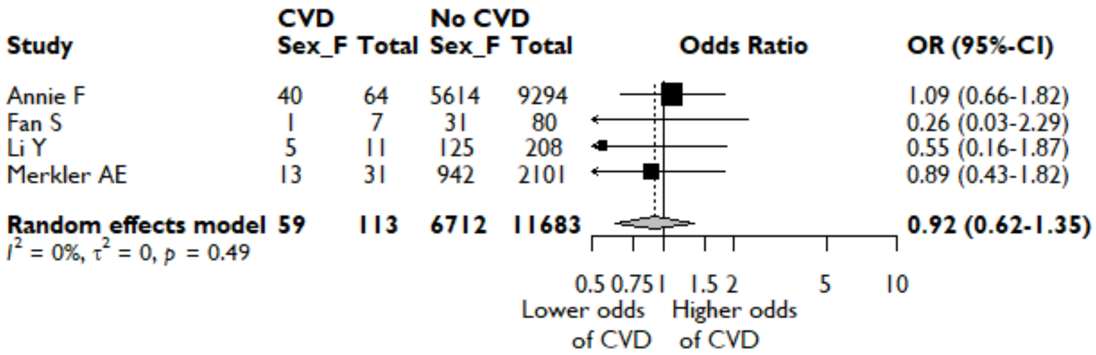

Figure 2b.

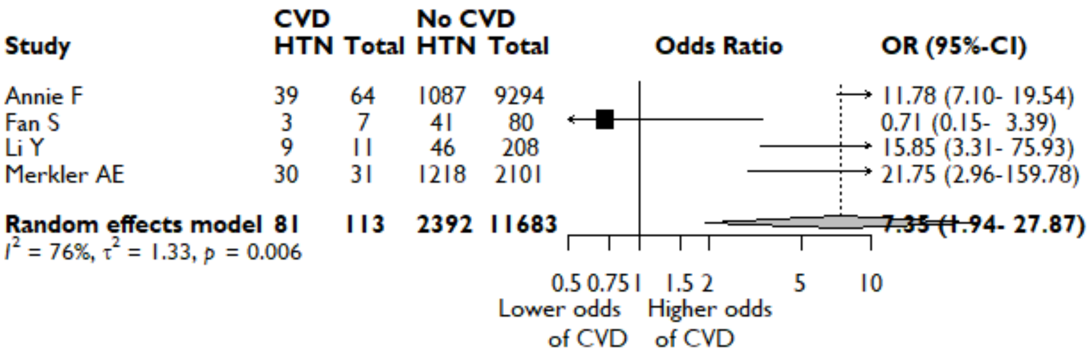

Figure 2c.

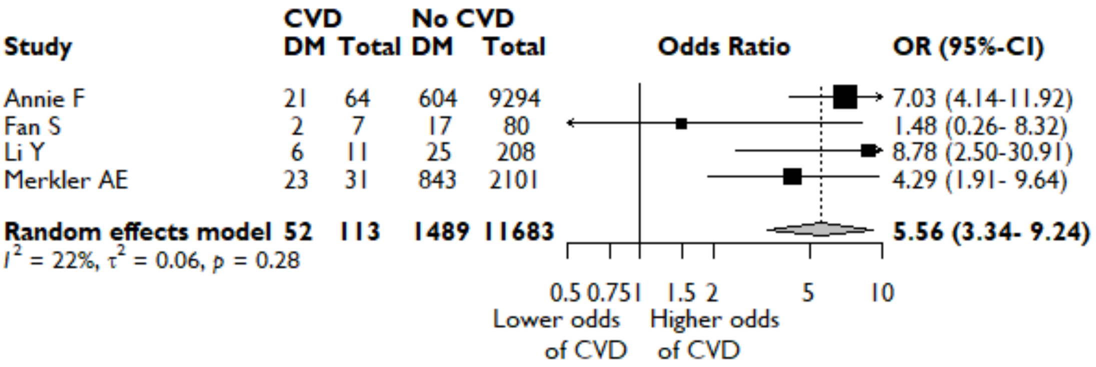

Figure 2d.

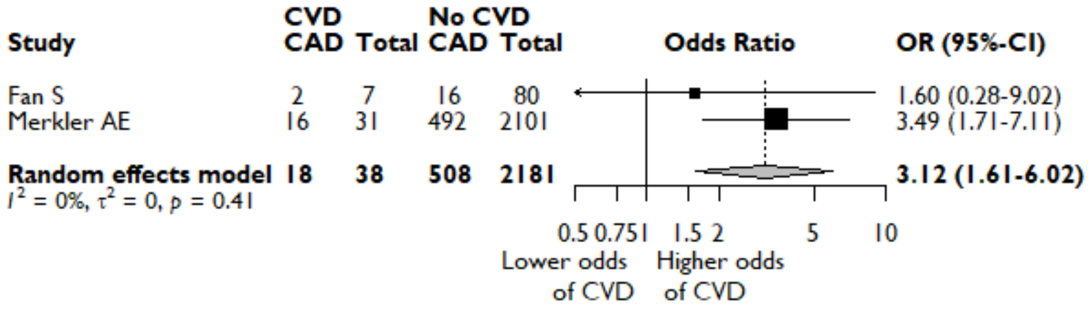

Figure 2e.

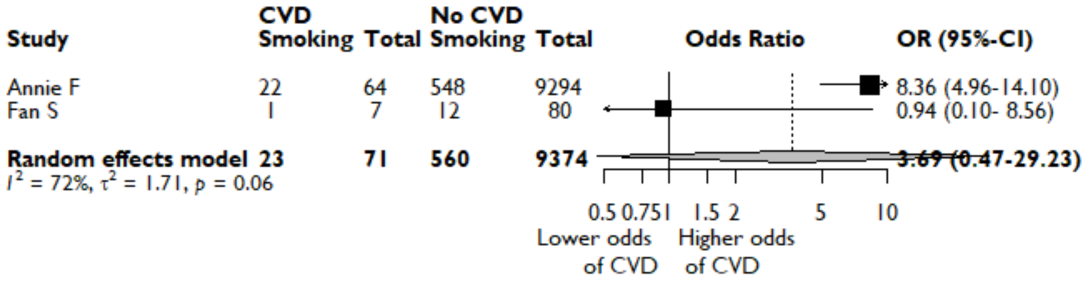

Figure 2f.

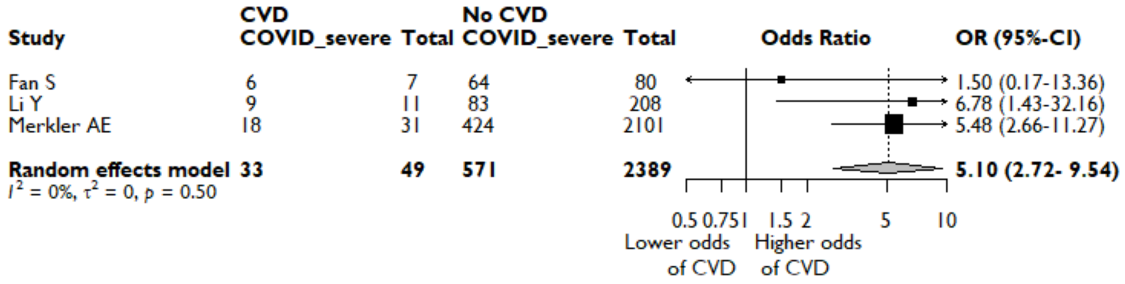

eFigure 3. Stroke etiologies among COVID-19 patients with stroke (Covid-19 group) and stroke patients without COVID-19 (No Covid-19 group), showing a significantly higher proportion of cryptogenic stroke in the Covid-19 group.

		Covid-19		No Covid-19			
**Stroke etiology**	**No. studies**	***Outcome***	***Total***	***Outcome***	***Total***	**OR (95%CI)**	**I^2**
Cardioembolic	2	9	41	49	177	0.72 (0.32-1.62)	0
Atherosclerosis	2	6	41	35	177	0.80 (0.31-2.04)	0
Small vessel disease	2	1	41	20	177	0.42 (0.04-4.07)	37
Cryptogenic	2	26	41	56	177	3.40 (1.16-10.00)	46

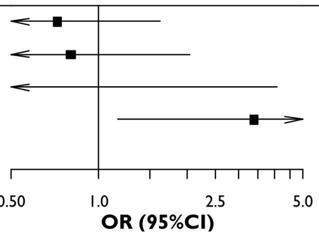

References:

1. Mandell LA, Wunderink RG, Anzueto A, Bartlett JG, Campbell GD, Dean NC, et al. Infectious Diseases Society of America/American Thoracic Society consensus guidelines on the management of community-acquired pneumonia in adults. Clin Infect Dis. 2007;44 Suppl 2:S27-72.
2. Higashida RT, Furlan AJ, Roberts H, Tomsick T, Connors B, Barr J, et al. Trial design and reporting standards for intra-arterial cerebral thrombolysis for acute ischemic stroke. Stroke. 2003;34(8):e109-e37.
3. Adams HP, Jr., Bendixen BH, Kappelle LJ, Biller J, Love BB, Gordon DL, et al. Classification of subtype of acute ischemic stroke. Definitions for use in a multicenter clinical trial. TOAST. Trial of Org 10172 in Acute Stroke Treatment. Stroke. 1993;24(1):35-41.
4. Agarwal A, Vishnu VY, Vibha D, Bhatia R, Gupta A, Das A, et al. Intracerebral Hemorrhage and SARS-CoV-2: Association or Causation. Ann Indian Acad Neurol. 2020;23(3):261-4.
5. Al Saiegh F, Ghosh R, Leibold A, Avery MB, Schmidt RF, Theofanis T, et al. Status of SARS-CoV-2 in cerebrospinal fluid of patients with COVID-19 and stroke. J Neurol Neurosurg Psychiatry. 2020;91(8):846-8.
6. Argirò R, Cirelli C, Sgreccia A, Del Sette B, Da Ros V. Cerebral hemorrhage related to vein thrombosis in Covid-19 patients in different Italian hospitals: View point for clinical and imaging implications. J Neurol Sci. 2020;416:117023.
7. Avula A, Nalleballe K, Narula N, Sapozhnikov S, Dandu V, Toom S, et al. COVID-19 presenting as stroke. Brain, Behavior, and Immunity. 2020;87:115-9.
8. Baldacini M, Pop R, Sattler L, Mauvieux L, Bilger K, Gantzer J, et al. Concomitant haemorrhagic syndrome and recurrent extensive arterial thrombosis in a patient with COVID-19 and acute promyelocytic leukaemia. British Journal of Haematology. 2020;189(6):1054-6.
9. Barrios-López JM, Rego-García I, Muñoz Martínez C, Romero-Fábrega JC, Rivero Rodríguez M, Ruiz Giménez JA, et al. Ischaemic stroke and SARS-CoV-2 infection: A causal or incidental association? Neurologia. 2020;35(5):295-302.
10. Baudar C, Duprez T, Kassab A, Miller N, Rutgers MP. COVID-19 as triggering co-factor for cortical cerebral venous thrombosis? J Neuroradiol. 2020.
11. Bigliardi G, Ciolli L, Giovannini G, Vandelli L, Dell'Acqua ML, Borzi GM, et al. Middle cerebral artery ischemic stroke and COVID-19: a case report. J Neurovirol. 2020.
12. Bolaji P, Kukoyi B, Ahmad N, Wharton C. Extensive cerebral venous sinus thrombosis: a potential complication in a patient with COVID-19 disease. BMJ Case Rep. 2020;13(8).
13. Bonardel C, Bonnerot M, Ludwig M, Vadot W, Beaune G, Chanzy B, et al. Bilateral Posterior Cerebral Artery Territory Infarction in a SARS-Cov-2 Infected Patient: discussion about an unusual case. Journal of Stroke and Cerebrovascular Diseases. 2020;29(9).
14. Bruggemann R, Gietema H, Jallah B, Ten Cate H, Stehouwer C, Spaetgens B. Arterial and venous thromboembolic disease in a patient with COVID-19: A case report. Thromb Res. 2020;191:153-5.
15. Burkert J, Patil S. Acute cerebrovascular event in a COVID-19 positive patient immediately after commencing non-invasive ventilation. BMJ Case Rep. 2020;13(9).
16. Carroll E, Lewis A. Catastrophic Intracranial Hemorrhage in Two Critically Ill Patients with COVID-19. Neurocrit Care. 2020:1-5.
17. Castillo PR, Del Brutto OH, Mautong H, García M, Tovar M, Middlebrooks EH, et al. Hemorrhagic stroke in hispanics with severe SARS-CoV2 infection. Journal of the Neurological Sciences. 2020;416.
18. Cavalcanti DD, Raz E, Shapiro M, Dehkharghani S, Yaghi S, Lillemoe K, et al. Cerebral Venous Thrombosis Associated with COVID-19. AJNR Am J Neuroradiol. 2020;41(8):1370-6.

19. Cavallieri F, Marti A, Fasano A, Salda AD, Ghirarduzzi A, Moratti C, et al. Prothrombotic state induced by COVID-19 infection as trigger for stroke in young patients: A dangerous association. eNeurologicalSci. 2020;20.
20. Chen W, Wang J, Cheng W, Li D, Zheng Y, Zhang J, et al. Hidden risk of nosocomial transmission: a presymptomatic novel coronavirus disease-19 (COVID-19) case with ischemic stroke. J Thorac Dis. 2020;12(6):3442-4.
21. Chibane S, Gibeau G, Poulin F, Tessier P, Goulet M, Carrier M, et al. Hyperacute multi-organ thromboembolic storm in COVID-19: a case report. J Thromb Thrombolysis. 2020:1-4.
22. Chougar L, Mathon B, Weiss N, Degos V, Shor N. Atypical Deep Cerebral Vein Thrombosis with Hemorrhagic Venous Infarction in a Patient Positive for COVID-19. AJNR Am J Neuroradiol. 2020;41(8):1377-9.
23. Co COC, Yu JRT, Laxamana LC, David-Ona DIA. Intravenous Thrombolysis for Stroke in a COVID-19 Positive Filipino Patient, a Case Report. J Clin Neurosci. 2020;77:234-6.
24. Deliwala S, Abdulhamid S, Abusalih MF, Al-Qasmi MM, Bachuwa G. Encephalopathy as the Sentinel Sign of a Cortical Stroke in a Patient Infected With Coronavirus Disease-19 (COVID-19). Cureus. 2020;12(5):e8121.
25. Diaz-Segarra N, Edmond A, Kunac A, Yonclas P. COVID-19 Ischemic Strokes as an Emerging Rehabilitation Population: A Case Series. Am J Phys Med Rehabil. 2020.
26. Duroi I, Van Durme F, Bruyns T, Louage S, Heyse A. Fatal Ischaemic Stroke During COVID-19 and Acute Lung Injury. Eur J Case Rep Intern Med. 2020;7(6):001732.
27. Fara MG, Stein LK, Skliut M, Morgello S, Fifi JT, Dhamoon MS. Macrothrombosis and stroke in patients with mild Covid-19 infection. J Thromb Haemost. 2020;18(8):2031-3.
28. Frisullo G, Bellavia S, Scala I, Piano C, Morosetti R, Brunetti V, et al. Stroke and COVID19: Not only a large-vessel disease. Journal of Stroke and Cerebrovascular Diseases. 2020;29(10).
29. Garaci F, Di Giuliano F, Picchi E, Da Ros V, Floris R. Venous cerebral thrombosis in COVID-19 patient. J Neurol Sci. 2020;414:116871.
30. Garg A, Marji A, Goyal S, Ismail R. A Case of COVID-19 With Memory Impairment and Delayed Presentation as Stroke. Cureus. 2020;12(8):e10025.
31. Gemcioglu E, Erden A, Davutoglu M, Karabuga B, Kucuksahin O. Acute Ischemic Stroke in a Lupus Anticoagulant-Positive Woman With COVID-19. J Clin Rheumatol. 2020.
32. Ghani MU, Kumar M, Ghani U, Sonia F, Abbas SA. Intracranial hemorrhage complicating anticoagulant prophylactic therapy in three hospitalized COVID-19 patients. J Neurovirol. 2020;26(4):602-4.
33. Gill I, Chan S, Fitzpatrick D. COVID-19-associated pulmonary and cerebral thromboembolic disease. Radiol Case Rep. 2020;15(8):1242-9.
34. Goldberg MF, Goldberg MF, Cerejo R, Tayal AH. Cerebrovascular Disease in COVID-19. AJNR Am J Neuroradiol. 2020;41(7):1170-2.
35. González-Pinto T, Luna-Rodríguez A, Moreno-Estébanez A, Agirre-Beitia G, Rodríguez-Antigüedad A, Ruiz-Lopez M. Emergency room neurology in times of COVID-19: malignant ischaemic stroke and SARS-CoV-2 infection. Eur J Neurol. 2020.
36. Griffin DO, Jensen A, Khan M, Chin J, Chin K, Parnell R, et al. Arterial thromboembolic complications in COVID-19 in low-risk patients despite prophylaxis. British Journal of Haematology. 2020;190(1):e11-e3.
37. Guillan M, Villacieros-Alvarez J, Bellido S, Perez-Jorge Peremarch C, Suarez-Vega VM, Aragones-Garcia M, et al. Unusual simultaneous cerebral infarcts in multiple arterial territories in a COVID-19 patient. Thrombosis Research. 2020;193:107-9.

38. Gulko E, Gomes W, Ali S, Al-Mufti F, Mehta H. Acute Common Carotid Artery Bifurcation Thrombus: An Emerging Pattern of Acute Strokes in Patients with COVID-19? AJNR Am J Neuroradiol. 2020;41(8):E65-e6.
39. Gunasekaran K, Amoah K, Rajasurya V, Buscher MG. Stroke in a young COVID-19 patient. Qjm. 2020;113(8):573-4.
40. Helms J, Tacquard C, Severac F, Leonard-Lorant I, Ohana M, Delabranche X, et al. High risk of thrombosis in patients with severe SARS-CoV-2 infection: a multicenter prospective cohort study. Intensive Care Medicine. 2020;46(6):1089-98.
41. Helms J, Kremer S, Merdji H, Clere-Jehl R, Schenck M, Kummerlen C, et al. Neurologic Features in Severe SARS-CoV-2 Infection. N Engl J Med. 2020;382(23):2268-70.
42. Heman-Ackah SM, Su YS, Spadola M, Petrov D, Chen HI, Schuster J, et al. Neurologically Devastating Intraparenchymal Hemorrhage in COVID-19 Patients on Extracorporeal Membrane Oxygenation: A Case Series. Neurosurgery. 2020;87(2):E147-e51.
43. Hemasian H, Ansari B. First case of Covid-19 presented with cerebral venous thrombosis: A rare and dreaded case. Rev Neurol (Paris). 2020;176(6):521-3.
44. Hoelscher C, Sweid A, Ghosh R, Al Saiegh F, Keppetipola KM, Farrell CJ, et al. Cerebral deep venous thrombosis and COVID-19: case report. J Neurosurg. 2020:1-4.
45. Hossri S, Shadi M, Hamarsha Z, Schneider R, El-Sayegh D. Clinically significant anticardiolipin antibodies associated with COVID-19. Journal of Critical Care. 2020;59:32-4.
46. Hughes C, Nichols T, Pike M, Subbe C, Elghenzai S. Cerebral Venous Sinus Thrombosis as a Presentation of COVID-19. Eur J Case Rep Intern Med. 2020;7(5):001691.
47. Jensen MP, Le Quesne J, Officer-Jones L, Teodosio A, Thaventhiran J, Ficken C, et al. Neuropathological findings in two patients with fatal COVID-19. Neuropathol Appl Neurobiol. 2020.
48. Kariyanna PT, Chandrakumar HP, Jayarangaiah A, Khan A, Vulkanov V, Ashamalla M, et al. Apical Takotsubo Cardiomyopathy in a COVID-19 Patient Presenting with Stroke: A Case Report and Pathophysiologic Insights. Am J Med Case Rep. 2020;8(10):350-7.
49. Khan AW, Ullah I, Khan KS. Ischemic stroke leading to bilateral vision loss in COVID-19 patient-A rare case report. J Med Virol. 2020.
50. Klein DE, Libman R, Kirsch C, Arora R. Cerebral venous thrombosis: A typical presentation of COVID-19 in the young. J Stroke Cerebrovasc Dis. 2020;29(8):104989.
51. Li J, Long X, Zhu C, Hu S, Lin Z, Li J, et al. A case of COVID-19 pneumonia with cerebral hemorrhage. Thromb Res. 2020;193:22-4.
52. Lima CFC, Holanda JLB, Pessoa MSL, Coimbra PPA. Acute ischemic stroke in a patient with COVID-19. Arq Neuropsiquiatr. 2020;78(7):454-5.
53. Llanso L, Urra X. Posterior Reversible Encephalopathy Syndrome in COVID-19 Disease: a Case-Report. SN Compr Clin Med. 2020:1-3.
54. Mahboob S, Boppana SH, Rose NB, Beutler BD, Tabaac BJ. Large vessel stroke and COVID-19: Case report and literature review. eNeurologicalSci. 2020;20:100250.
55. Maldonado Slootjes S, Hammer F, Leempoel J, Peeters A. Acute stroke care during the COVID-19 pandemic: difficult, but not impossible! Acta Neurol Belg. 2020;120(5):1257-8.
56. Malentacchi M, Gned D, Angelino V, Demichelis S, Perboni A, Veltri A, et al. Concomitant brain arterial and venous thrombosis in a COVID-19 patient. Eur J Neurol. 2020.
57. Moshayedi P, Ryan TE, Mejia LLP, Nour M, Liebeskind DS. Triage of Acute Ischemic Stroke in Confirmed COVID-19: Large Vessel Occlusion Associated With Coronavirus Infection. Front Neurol. 2020;11:353.
58. Motoie R, Akai M, Kitahara T, Imamura H, Tanabe T, Sarazawa K, et al. Coronavirus Disease 2019 Complicated by Multiple Simultaneous Intracerebral Hemorrhages. Intern Med. 2020.

59. Muhammad S, Petridis A, Cornelius JF, Hänggi D. Letter to editor: Severe brain haemorrhage and concomitant COVID-19 Infection: A neurovascular complication of COVID-19. Brain, Behavior, and Immunity. 2020;87:150-1.
60. Nicholson P, Alshafai L, Krings T. Neuroimaging findings in patients with COVID-19. American Journal of Neuroradiology. 2020;41(8).
61. Papi C, Spagni G, Alexandre A, Calabresi P, Della Marca G, Broccolini A. Unprotected stroke management in an undiagnosed case of Severe Acute Respiratory Syndrome Coronavirus 2 infection. J Stroke Cerebrovasc Dis. 2020;29(9):104981.
62. Poillon G, Obadia M, Perrin M, Savatovsky J, Lecler A. Cerebral venous thrombosis associated with COVID-19 infection: Causality or coincidence? J Neuroradiol. 2020.
63. Radmard S, Epstein SE, Roeder HJ, Michalak AJ, Shapiro SD, Boehme A, et al. Inpatient Neurology Consultations During the Onset of the SARS-CoV-2 New York City Pandemic: A Single Center Case Series. Frontiers in Neurology. 2020;11.
64. Rinkel LA, Prick JCM, Slot RER, Sombroek NMA, Burggraaff J, Groot AE, et al. Impact of the COVID-19 outbreak on acute stroke care. J Neurol. 2020:1-6.
65. Roy-Gash F, Marine M, Jean-Michel D, Herve V, Raphael B, Nicolas E. COVID-19-associated acute cerebral venous thrombosis: clinical, CT, MRI and EEG features. Crit Care. 2020;24(1):419.
66. Rudilosso S, Esteller D, Urra X, Chamorro Á. Thalamic perforating artery stroke on computed tomography perfusion in a patient with coronavirus disease 2019. J Stroke Cerebrovasc Dis. 2020;29(8):104974.
67. Saggese CE, Del Bianco C, Di Ruzza MR, Magarelli M, Gandini R, Plocco M. COVID-19 and Stroke: Casual or Causal Role? Cerebrovasc Dis. 2020;49(3):341-4.
68. Salahuddin H, Castonguay AC, Zaidi SF, Burgess R, Jadhav AP, Jumaa MA. Interventional Stroke Care in the Era of COVID-19. Front Neurol. 2020;11:468.
69. Sangalli D, Polonia V, Colombo D, Mantero V, Filizzolo M, Scaccabarozzi C, et al. A single-centre experience of intravenous thrombolysis for stroke in COVID-19 patients. Neurol Sci. 2020;41(9):2325-9.
70. Scullen T, Keen J, Mathkour M, Dumont AS, Kahn L. Coronavirus 2019 (COVID-19)-Associated Encephalopathies and Cerebrovascular Disease: The New Orleans Experience. World Neurosurg. 2020.
71. Seabra C, Silva B, Fagundes V, Rocha J, Nogueira L, Mesquita M. Should the Management of Embolic Stroke in the Elderly Be Changed if They Also Have COVID-19? Eur J Case Rep Intern Med. 2020;7(6):001736.
72. Sharifi-Razavi A, Karimi N, Rouhani N. COVID-19 and intracerebral haemorrhage: causative or coincidental? New Microbes New Infect. 2020;35:100669.
73. Sharifi-Razavi A, Karimi N, Zarvani A, Cheraghmakani H, Baghbanian SM. Ischemic stroke associated with novel coronavirus 2019: a report of three cases. Int J Neurosci. 2020:1-5.
74. Sparr SA, Bieri PL. Infarction of the Splenium of the Corpus Callosum in the Age of COVID-19: A Snapshot in Time. Stroke. 2020:Strokeaha120030434.
75. Trifan G, Hillmann M, Testai FD. Acute Stroke as the Presenting Symptom of SARS-CoV-2 Infection in a Young Patient with Cerebral Autosomal Dominant Arteriopathy with Subcortical Infarcts and Leukoencephalopathy. Journal of Stroke and Cerebrovascular Diseases. 2020;29(10).
76. TunÇ A, ÜnlÜbaŞ Y, Alemdar M, AkyÜz E. Coexistence of COVID-19 and acute ischemic stroke report of four cases. J Clin Neurosci. 2020;77:227-9.
77. Usman AA, Han J, Acker A, Olia SE, Bermudez C, Cucchiara B, et al. A Case Series of Devastating Intracranial Hemorrhage During Venovenous Extracorporeal Membrane Oxygenation for COVID-19. J Cardiothorac Vasc Anesth. 2020;34(11):3006-12.

78. Valderrama EV, Humbert K, Lord A, Frontera J, Yaghi S. Severe Acute Respiratory Syndrome Coronavirus 2 Infection and Ischemic Stroke. Stroke. 2020;51(7):e124-e7.
79. Viguier A, Delamarre L, Duplantier J, Olivot JM, Bonneville F. Acute ischemic stroke complicating common carotid artery thrombosis during a severe COVID-19 infection. J Neuroradiol. 2020;47(5):393-4.
80. Vu D, Ruggiero M, Choi WS, Masri D, Flyer M, Shyknevsky I, et al. Three unsuspected CT diagnoses of COVID-19. Emerg Radiol. 2020;27(3):229-32.
81. Wee NK, Fan EB, Lee KCH, Chia YW, Lim TCC. CT Fluid-Blood Levels in COVID-19 Intracranial Hemorrhage. AJNR Am J Neuroradiol. 2020.
82. Williams OH, Mohideen S, Sen A, Martinovic O, Hart J, Brex PA, et al. Multiple internal border zone infarcts in a patient with COVID-19 and CADASIL. Journal of the Neurological Sciences. 2020;416.
83. Zahid MJ, Baig A, Galvez-Jimenez N, Martinez N. Hemorrhagic stroke in setting of severe COVID-19 infection requiring Extracorporeal Membrane Oxygenation (ECMO). J Stroke Cerebrovasc Dis. 2020;29(9):105016.
84. Zayet S, Klopfenstein T, Kovȧcs R, Stancescu S, Hagenkötter B. Acute Cerebral Stroke with Multiple Infarctions and COVID-19, France, 2020. Emerg Infect Dis. 2020;26(9).
85. Zhai P, Ding Y, Li Y. The impact of COVID-19 on ischemic stroke. Diagnostic Pathology. 2020;15(1).
86. Zhang Y, Xiao M, Zhang S, Xia P, Cao W, Jiang W, et al. Coagulopathy and Antiphospholipid Antibodies in Patients with Covid-19. N Engl J Med. 2020;382(17):e38.
87. Zhou B, She J, Wang Y, Ma X. A Case of Coronavirus Disease 2019 With Concomitant Acute Cerebral Infarction and Deep Vein Thrombosis. Front Neurol. 2020;11:296.
88. Al Kasab S, Almallouhi E, Alawieh A, Levitt MR, Jabbour P, Sweid A, et al. International experience of mechanical thrombectomy during the COVID-19 pandemic: insights from STAR and ENRG. J NeuroInterv Surg. 2020:neurintsurg-2020-016671.
89. Altschul DJ, Esenwa C, Haranhalli N, Unda SR, de La Garza Ramos R, Dardick J, et al. Predictors of mortality for patients with COVID-19 and large vessel occlusion. Interv Neuroradiol 0(0):1591019920954603.
90. Altschul DJ, Unda SR, de La Garza Ramos R, Zampolin R, Benton J, Holland R, et al. Hemorrhagic presentations of COVID-19: Risk factors for mortality. Clin Neurol Neurosurg. 2020;198:106112.
91. Annie F, Bates MC, Nanjundappa A, Bhatt DL, Alkhouli M. Prevalence and Outcomes of Acute Ischemic Stroke Among Patients ≤50 Years of Age With Laboratory Confirmed COVID-19 Infection. Am J Cardiol. 2020.
92. Ashrafi F, Zali A, Ommi D, Salari M, Fatemi A, Arab-Ahmadi M, et al. COVID-19-related strokes in adults below 55 years of age: a case series. Neurological Sciences. 2020;41(8):1985-9.
93. Belani P, Schefflein J, Kihira S, Rigney B, Delman BN, Mahmoudi K, et al. COVID-19 Is an Independent Risk Factor for Acute Ischemic Stroke. AJNR Am J Neuroradiol. 2020;41(8):1361-4.
94. Benger M, Williams O, Siddiqui J, Sztriha L. Intracerebral haemorrhage and COVID-19: Clinical characteristics from a case series. Brain Behav Immun. 2020;88:940-4.
95. Benussi A, Pilotto A, Premi E, Libri I, Giunta M, Agosti C, et al. Clinical characteristics and outcomes of inpatients with neurologic disease and COVID-19 in Brescia, Lombardy, Italy. Neurology. 2020;95(7):e910-e20.
96. Berekashvili K, Dmytriw AA, Vulkanov V, Agarwal S, Khaneja A, Turkel-Parella D, et al. Etiologic Subtypes of Ischemic Stroke in SARS-COV-2 Virus patients. medRxiv. 2020:2020.05.03.20077206.

97. Beyrouti R, Adams ME, Benjamin L, Cohen H, Farmer SF, Goh YY, et al. Characteristics of ischaemic stroke associated with COVID-19. J Neurol Neurosurg Psychiatry. 2020;91(8):889-91.
98. Cantador E, Núñez A, Sobrino P, Espejo V, Fabia L, Vela L, et al. Incidence and consequences of systemic arterial thrombotic events in COVID-19 patients. J Thromb Thrombolysis. 2020:1-5.
99. Cappellari M, Zini A, Sangalli D, Cavallini A, Reggiani M, Nicoletta Sepe F, et al. Thrombolysis and bridging therapy in patients with acute ischemic stroke and Covid-19. Eur J Neurol.n/a(n/a).
100. Chougar L, Shor N, Weiss N, Galanaud D, Leclercq D, Mathon B, et al. Retrospective Observational Study of Brain Magnetic Resonance Imaging Findings in Patients with Acute SARS-CoV-2 Infection and Neurological Manifestations. Radiology. 2020:202422.
101. D'Amore F, Vinacci G, Agosti E, Cariddi LP, Terrana AV, Vizzari FA, et al. Pressing Issues in COVID-19: Probable Cause to Seize SARS-CoV-2 for Its Preferential Involvement of Posterior Circulation Manifesting as Severe Posterior Reversible Encephalopathy Syndrome and Posterior Strokes. AJNR Am J Neuroradiol. 2020.
102. D'Anna L, Kwan J, Brown Z, Halse O, Jamil S, Kalladka D, et al. Characteristics and clinical course of Covid-19 patients admitted with acute stroke. J Neurol. 2020:1-5.
103. Dmytriw AA, Phan K, Schirmer C, Settecase F, Heran MKS, Efendizade A, et al. Ischaemic stroke associated with COVID-19 and racial outcome disparity in North America. J Neurol Neurosurg Psychiatry. 2020.
104. Dogra S, Jain R, Cao M, Bilaloglu S, Zagzag D, Hochman S, et al. Hemorrhagic stroke and anticoagulation in COVID-19. J Stroke Cerebrovasc Dis. 2020;29(8):104984.
105. Escalard S, Maïer B, Redjem H, Delvoye F, Hébert S, Smajda S, et al. Treatment of Acute Ischemic Stroke due to Large Vessel Occlusion With COVID-19: Experience From Paris. Stroke. 2020;51(8):2540-3.
106. Fan S, Xiao M, Han F, Xia P, Bai X, Chen H, et al. Neurological Manifestations in Critically Ill Patients With COVID-19: A Retrospective Study. Front Neurol. 2020;11:806.
107. Hernández-Fernández F, Valencia HS, Barbella-Aponte RA, Collado-Jiménez R, Ayo-Martín Ó, Barrena C, et al. Cerebrovascular disease in patients with COVID-19: neuroimaging, histological and clinical description. Brain. 2020.
108. Immovilli P, Terracciano C, Zaino D, Marchesi E, Morelli N, Terlizzi E, et al. Stroke in COVID-19 patients-A case series from Italy. Int J Stroke. 2020;15(6):701-2.
109. Jain R, Young M, Dogra S, Kennedy H, Nguyen V, Jones S, et al. COVID-19 related neuroimaging findings: A signal of thromboembolic complications and a strong prognostic marker of poor patient outcome. J Neurol Sci. 2020;414.
110. Jillella DV, Janocko NJ, Nahab F, Benameur K, Greene JG, Wright WL, et al. Ischemic Stroke in COVID-19: An Urgent Need for Early Identification and Management. medRxiv. 2020:2020.05.25.20111047.
111. John S, Kesav P, Mifsud VA, Piechowski-Jozwiak B, Dibu J, Bayrlee A, et al. Characteristics of Large-Vessel Occlusion Associated with COVID-19 and Ischemic Stroke. AJNR Am J Neuroradiol. 2020.
112. Karadaş Ö, Öztürk B, Sonkaya AR. A prospective clinical study of detailed neurological manifestations in patients with COVID-19. Neurological Sciences. 2020;41(8):1991-5.
113. Katz JM, Libman RB, Wang JJ, Sanelli P, Filippi CG, Gribko M, et al. Cerebrovascular Complications of COVID-19. Stroke. 2020:Strokeaha120031265.
114. Khan M, Ibrahim RH, Siddiqi SA, Kerolos Y, Al-Kaylani MM, AlRukn SA, et al. COVID-19 and acute ischemic stroke - A case series from Dubai, UAE. Int J Stroke. 2020;15(6):699-700.

115. Kihira S, Schefflein J, Mahmoudi K, Rigney B, B ND, Mocco J, et al. Association of Coronavirus Disease (COVID-19) With Large Vessel Occlusion Strokes: A Case-Control Study. AJR Am J Roentgenol. 2020:1-6.
116. Klok FA, Kruip M, van der Meer NJM, Arbous MS, Gommers D, Kant KM, et al. Confirmation of the high cumulative incidence of thrombotic complications in critically ill ICU patients with COVID-19: An updated analysis. Thromb Res. 2020;191:148-50.
117. Kremer S, Lersy F, Anheim M, Merdji H, Schenck M, Oesterlé H, et al. Neurologic and neuroimaging findings in COVID-19 patients: A retrospective multicenter study. Neurology. 2020.
118. Kvernland A, Kumar A, Yaghi S, Raz E, Frontera J, Lewis A, et al. Anticoagulation use and Hemorrhagic Stroke in SARS-CoV-2 Patients Treated at a New York Healthcare System. Neurocritical Care. 2020.
119. Lapergue B, Lyoubi A, Meseguer E, Avram I, Denier C, Venditti L, et al. Large vessel stroke in six patients following SARS-CoV-2 infection: a retrospective case study series of acute thrombotic complications on stable underlying atherosclerotic disease. Eur J Neurol. 2020.
120. Li Y, Li M, Wang M, Zhou Y, Chang J, Xian Y, et al. Acute cerebrovascular disease following COVID-19: a single center, retrospective, observational study. Stroke Vasc Neurol. 2020.
121. Liang JW, Reynolds AS, Reilly K, Lay C, Kellner CP, Shigematsu T, et al. COVID-19 and Decompressive Hemicraniectomy for Acute Ischemic Stroke. Stroke. 2020:Strokeaha120030804.
122. Lin C, Arevalo YA, Nanavati HD, Lin DM. Racial differences and an increased systemic inflammatory response are seen in patients with COVID-19 and ischemic stroke. Brain Behav Immun Health. 2020;8:100137.
123. Lodigiani C, Iapichino G, Carenzo L, Cecconi M, Ferrazzi P, Sebastian T, et al. Venous and arterial thromboembolic complications in COVID-19 patients admitted to an academic hospital in Milan, Italy. Thromb Res. 2020;191:9-14.
124. Majidi S, Fifi JT, Ladner TR, Lara-Reyna J, Yaeger KA, Yim B, et al. Emergent Large Vessel Occlusion Stroke During New York City's COVID-19 Outbreak: Clinical Characteristics and Paraclinical Findings. Stroke. 2020:Strokeaha120030397.
125. Mao L, Jin H, Wang M, Hu Y, Chen S, He Q, et al. Neurologic Manifestations of Hospitalized Patients With Coronavirus Disease 2019 in Wuhan, China. JAMA Neurol. 2020;77(6):1-9.
126. Mehrpour M, Shuaib A, Farahani M, Hatamabadi H, Fatehi Z, Ghaffari M, et al. Coronavirus disease 2019 and stroke in Iran: a case series and effects on stroke admissions. Int J Stroke.0(0):1747493020937397.
127. Meppiel E, Peiffer-Smadja N, Maury A, Bekri I, Delorme C, Desestret V, et al. Neurological manifestations associated with COVID-19: a nationwide registry. medRxiv. 2020:2020.07.15.20154260.
128. Merkler AE, Parikh NS, Mir S, Gupta A, Kamel H, Lin E, et al. Risk of Ischemic Stroke in Patients with Covid-19 versus Patients with Influenza. medRxiv. 2020.
129. Mohamud AY, Griffith B, Rehman M, Miller D, Chebl A, Patel SC, et al. Intraluminal Carotid Artery Thrombus in COVID-19: Another Danger of Cytokine Storm? AJNR Am J Neuroradiol. 2020.
130. Morassi M, Bagatto D, Cobelli M, D'Agostini S, Gigli GL, Bnà C, et al. Stroke in patients with SARS-CoV-2 infection: case series. J Neurol. 2020;267(8):2185-92.
131. Nalleballe K, Reddy Onteddu S, Sharma R, Dandu V, Brown A, Jasti M, et al. Spectrum of neuropsychiatric manifestations in COVID-19. Brain Behav Immun. 2020;88:71-4.
132. Nawabi J, Morotti A, Wildgruber M, Boulouis G, Kraehling H, Schlunk F, et al. Clinical and Imaging Characteristics in Patients with SARS-CoV-2 Infection and Acute Intracranial Hemorrhage. J Clin Med. 2020;9(8).

133. Ntaios G, Michel P, Georgiopoulos G, Guo Y, Li W, Xiong J, et al. Characteristics and Outcomes in Patients With COVID-19 and Acute Ischemic Stroke: The Global COVID-19 Stroke Registry. Stroke. 2020;51(9):e254-e8.
134. Oxley TJ, Mocco J, Majidi S, Kellner CP, Shoirah H, Singh IP, et al. Large-Vessel Stroke as a Presenting Feature of Covid-19 in the Young. N Engl J Med. 2020;382(20):e60.
135. Paterson RW, Brown RL, Benjamin L, Nortley R, Wiethoff S, Bharucha T, et al. The emerging spectrum of COVID-19 neurology: clinical, radiological and laboratory findings. Brain. 2020.
136. Pinna P, Grewal P, Hall JP, Tavarez T, Dafer RM, Garg R, et al. Neurological manifestations and COVID-19: Experiences from a tertiary care center at the Frontline. J Neurol Sci. 2020;415:116969.
137. Pons-Escoda A, Naval-Baudín P, Majós C, Camins A, Cardona P, Cos M, et al. Neurologic Involvement in COVID-19: Cause or Coincidence? A Neuroimaging Perspective. AJNR Am J Neuroradiol. 2020;41(8):1365-9.
138. Pop R, Hasiu A, Bolognini F, Mihoc D, Quenardelle V, Gheoca R, et al. Stroke Thrombectomy in Patients with COVID-19: Initial Experience in 13 Cases. AJNR Am J Neuroradiol. 2020.
139. Reddy ST, Garg T, Shah C, Nascimento FA, Imran R, Kan P, et al. Cerebrovascular Disease in Patients with COVID-19: A Review of the Literature and Case Series. Case Rep Neurol. 2020;12(2):199-209.
140. Rothstein A, Oldridge O, Schwennesen H, Do D, Cucchiara BL. Acute Cerebrovascular Events in Hospitalized COVID-19 Patients. Stroke. 2020:Strokeaha120030995.
141. Shahjouei S, Naderi S, Li J, Khan A, Chaudhary D, Farahmand G, et al. Risk of stroke in hospitalized SARS-CoV-2 infected patients: A multinational study. EBioMedicine. 2020;59:102939.
142. Siegler JE, Cardona P, Arenillas JF, Talavera B, Nunez Guillen A, Chavarria-Miranda A, et al. EXPRESS: Cerebrovascular events and outcomes in hospitalized patients with COVID-19: The SVIN COVID-19 Multinational Registry. Int J Stroke. 2020:1747493020959216.
143. Sweid A, Hammoud B, Bekelis K, Missios S, Tjoumakaris SI, Gooch MR, et al. Cerebral ischemic and hemorrhagic complications of coronavirus disease 2019. Int J Stroke. 2020:1747493020937189.
144. Varatharaj A, Thomas N, Ellul MA, Davies NWS, Pollak TA, Tenorio EL, et al. Neurological and neuropsychiatric complications of COVID-19 in 153 patients: a UK-wide surveillance study. Lancet Psychiatry. 2020.
145. Wang A, Mandigo GK, Yim PD, Meyers PM, Lavine SD. Stroke and mechanical thrombectomy in patients with COVID-19: Technical observations and patient characteristics. J NeuroInterv Surg. 2020;12(7):648-53.
146. Xiong W, Mu J, Guo J, Lu L, Liu D, Luo J, et al. New onset neurologic events in people with COVID-19 infection in three regions in China. Neurology. 2020.
147. Yaeger KA, Fifi JT, Lara-Reyna J, Rossitto C, Ladner T, Yim B, et al. Initial Stroke Thrombectomy Experience in New York City during the COVID-19 Pandemic. AJNR Am J Neuroradiol. 2020;41(8):1357-60.
148. Yaghi S, Ishida K, Torres J, Mac Grory B, Raz E, Humbert K, et al. SARS-CoV-2 and Stroke in a New York Healthcare System. Stroke. 2020;51(7):2002-11.
